# Founder events influence structures of *Aspergillus flavus populations*

**DOI:** 10.1111/1462-2920.15122

**Published:** 2020-06-27

**Authors:** Alejandro Ortega‐Beltran, Kenneth A. Callicott, Peter J. Cotty

**Affiliations:** ^1^ School of Plant Sciences University of Arizona Tucson AZ 85721 USA; ^2^ USDA‐ARS Tucson AZ 85721 USA; ^3^ International Institute of Tropical Agriculture PMB 5320 Oyo Road, Ibadan Nigeria; ^4^ School of Food Science and Engineering Ocean University of China Qingdao Shandong 266003 China

## Abstract

In warm regions, agricultural fields are occupied by complex *Aspergillus flavus* communities composed of isolates in many vegetative compatibility groups (VCGs) with varying abilities to produce highly toxic, carcinogenic aflatoxins. Aflatoxin contamination is reduced with biocontrol products that enable atoxigenic isolates from atoxigenic VCGs to dominate the population. Shifts in VCG frequencies similar to those caused by the introduction of biocontrol isolates were detected in Sonora, Mexico, where biocontrol is not currently practiced. The shifts were attributed to founder events. Although VCGs reproduce clonally, significant diversity exists within VCGs. Simple sequence repeat (SSR) fingerprinting revealed that increased frequencies of VCG YV150 involved a single haplotype. This is consistent with a founder event. Additionally, great diversity was detected among 82 YV150 isolates collected over 20 years across Mexico and the United States. Thirty‐six YV150 haplotypes were separated into two populations by Structure and SplitsTree analyses. Sixty‐five percent of isolates had *MAT1‐1* and belonged to one population. The remaining had *MAT1‐2* and belonged to the second population. SSR alleles varied within populations, but recombination between populations was not detected despite co‐occurrence at some locations. Results suggest that YV150 isolates with opposite mating‐type have either strongly restrained or lost sexual reproduction among themselves.

## Introduction

In warm agricultural areas, fungi belonging to *Aspergillus* section *Flavi* frequently infect and contaminate maize with aflatoxins (Cotty *et al*., 1994; Probst *et al*., 2010). Aflatoxins are naturally occurring, highly toxic and carcinogenic mycotoxins that can induce acute and/or chronic health detriments, including death, to both humans and animals (Probst *et al*., 2012; Monson *et al*., 2015; Xu *et al*., 2018). The most prevalent and toxic aflatoxin, B_1_, is classified as a Group 1 carcinogen by the International Agency for Research on Cancer (IARC, 2002). Aflatoxin B_1_ is strictly regulated in most developed nations (FAO, 2004; Wu and Khlangwiset, 2010), with the enforcement of these regulations limiting commercialization and consumption of contaminated commodities. These limitations may cause a severe economic loss for farmers, processors and consumers (Wu and Khlangwiset, 2010; Bui‐Klimke *et al*., 2014). However, in nations where regulations are either poorly enforced or non‐existent, humans and domestic animals are continuously exposed to aflatoxins (Gong *et al*., 2008; Shephard, 2008; Hoffmann *et al*., 2018; Voth‐Gaeddert *et al*., 2018). This results in losses in human health and livestock productivity that have a proportionately greater economic impact than that seen in the developed world (Udomkun *et al*., 2017; Nishimwe *et al*., 2019).

Aflatoxin‐producing fungi interacting with various crops are highly diverse in terms of community structure at the species and genotype level, toxin production potentials and adaptation to diverse cropping systems (Mehl *et al*., 2012). The most studied species is *A*. *flavus* because this is the most common causal agent of aflatoxin contamination (Cotty *et al*., 1994; Klich, 2007). *Aspergillus flavus* has two prominent morphotypes, the S and L, which differ in both morphology and physiology. The S morphotype produces numerous, small sclerotia (diameter < 400 μm) and consistently high levels of aflatoxin B_1_, while the L morphotype produces fewer, larger sclerotia (diameter > 400 μm) and variable levels of aflatoxin B_1_ (Cotty, 1989; Probst *et al*., 2012; Singh and Cotty, 2019). Variation in the aflatoxin‐producing potential of fungal communities has a large impact on the extent to which crops become contaminated (Probst *et al*., 2007; Cotty *et al*., 2008; Bandyopadhyay *et al*., 2016).

Each *A*. *flavus* morphotype is subdivided into numerous vegetative compatibility groups (VCGs). Members of the same VCG are isogenic for all vegetative incompatibility (*vic*) loci and form heterokaryons when their physiologically complementary hyphae anastomose (Bayman and Cotty, 1991; Leslie, 1993). Membership in a VCG is typically determined with functional vegetative compatibility analyses (VCA) using nitrate non‐utilizing (*nit*) mutants (Bayman and Cotty, 1991, 1993). Within each of the four *A*. *flavus* VCGs previously examined, variation among isolates is best explained by clonal descent with mutation over large numbers of generations (Grubisha and Cotty, 2010, 2015). For other *A*. *flavus* VCGs, members are more closely related to each other than to individuals within other VCGs (Papa, 1986; Bayman and Cotty, 1993).

*Aspergillus* is an anamorph genus, and *A*. *flavus* has been considered an asexual species (Papa, 1986; Cotty *et al*., 1994; Klich, 2007; Islam *et al*., 2018). However, functional mating‐type idiomorphs of *A*. *flavus* have been characterized (Ramirez‐Prado *et al*., 2008), and the possibility of sexual recombination under field conditions suggested following induction of low fertility ascomata under laboratory conditions between isolates of opposite mating‐type and different VCGs (Horn *et al*., 2009a, 2009b, 2014; Olarte *et al*., 2012). In addition, sexual reproduction in sclerotia may occur in the field if sclerotia incubated for 4 months in the laboratory are allowed to come in contact with natural *A*. *flavus* populations in the field (Horn *et al*., 2016). Those findings were detected in experiments conducted in fields in Georgia, USA. On the other hand, in agricultural fields in Arizona and Texas, there is no detectable recombination among sympatric VCGs co‐existing across extensive areas (Grubisha and Cotty, 2010). Results from studies examining aflatoxin‐producing fungal communities associated with maize in Mexico (Ortega‐Beltran *et al*., 2016), Kenya (Islam *et al*., 2018) and in several nations in sub‐Saharan Africa (Adhikari *et al*., 2016) suggest communities of aflatoxin‐producing fungi are (i) highly stable, (ii) shaped predominantly by clonal reproduction and mutation, and (iii) participate in little or no sexual recombination. The frequency of sexual recombination within *A*. *flavus* populations in nature remains unknown (Fedorova *et al*., 2009; Horn *et al*., 2009a, 2011, 2016; Kwon‐Chung and Sugui, 2009; Dyer and O'Gorman, 2012).

In a 3‐year study of *A*. *flavus* L morphotype populations associated with maize production in Sonora, Mexico, 136 VCGs were identified (Ortega‐Beltran and Cotty, 2018). However, VCG composition varied by year and no VCG was consistently dominant across years. Certain VCGs had drastic spatio‐temporal ‘shifts’, that is, had drastic changes in frequencies over single years in both multiple fields and multiple agro‐ecosystems. The most prominent shift was a change in the frequency of VCG SON003 (Ortega‐Beltran and Cotty, 2018). Subsequently, VCG SON003 was identified as previously described YV150, and SON003 is now referred to as YV150 (Mehl and Cotty, 2010) in the current work. YV150, originally characterized from cotton field soil in Yuma Valley, Arizona, in 1987, has been collected for over 20 years from crops and soils across an extensive geographic area ranging from Nayarit, Mexico to Arkansas, USA (South to North) and from Arizona to Georgia, USA (West to East). Variability for genetic markers among members of YV150 has not been previously described. The increase in the relative frequency of YV150 observed in 2006 in Sonora was hypothesized to be a result of a founder event in which genetic variation was lost when a small number of individuals from the larger population disproportionately established the population that developed the previous summer (Ortega‐Beltran and Cotty, 2018). However, no genetic evidence to test this hypothesis was developed and it remains unclear if shifts in YV150 frequencies in Sonora resulted from rapid increases of one or a few YV150 clones or a sudden increase by the VCG as a whole. In the latter case, the diversity of YV150 in 2006 would reflect the overall diversity of YV150. The causes for dominance and subsequent drastic decline of YV150 in Sonora, Mexico, also are unknown. However, the adaptability of members of VCG YV150 is reflected in repeated detection of this VCG for over 20 years and the frequent association of members of the VCG with maize and cotton production in North America.

The objectives of the present work were (i) to assess genetic diversity within 82 isolates of VCG YV150 from Mexico and the United States collected over 20 years using 24 microsatellite markers for *A*. *flavus* previously shown to be useful for quantifying variability within a VCG (Grubisha and Cotty, 2010, 2015; Ortega‐Beltran *et al*., 2016) and (ii) to determine if members of this VCG from spatially and temporally distinct populations possess clonal or recombinant population structures. The evidence gathered suggests swings in incidences of VCGs reflect influences of founder events. In addition, VCG YV150 was found to be similar to previously examined VCGs (Grubisha and Cotty, 2010, 2015; Ortega‐Beltran *et al*., 2016) in the diversity of SSR haplotypes but distinct in having the diversity distributed across isolates containing one or the other mating‐type idiomorph. Another important finding is that YV150 is composed of two divergent populations, one containing the *MAT1‐1* idiomorph and the other *MAT1‐2*. Previously it was emphasized that distributions of *A*. *flavus* VCGs are best described from samples collected over multiple years representing multiple sites distributed across large areas (Ortega‐Beltran *et al*., 2015). Dominance by a single haplotype of a VCG during 1 year, across a large area, in the presence of multiple haplotypes of the same VCG reported in the current work could not have been detected with less intensive sampling.

## Results

### Mating‐type idiomorph characterization in VCG YV150

**Fig 1:**
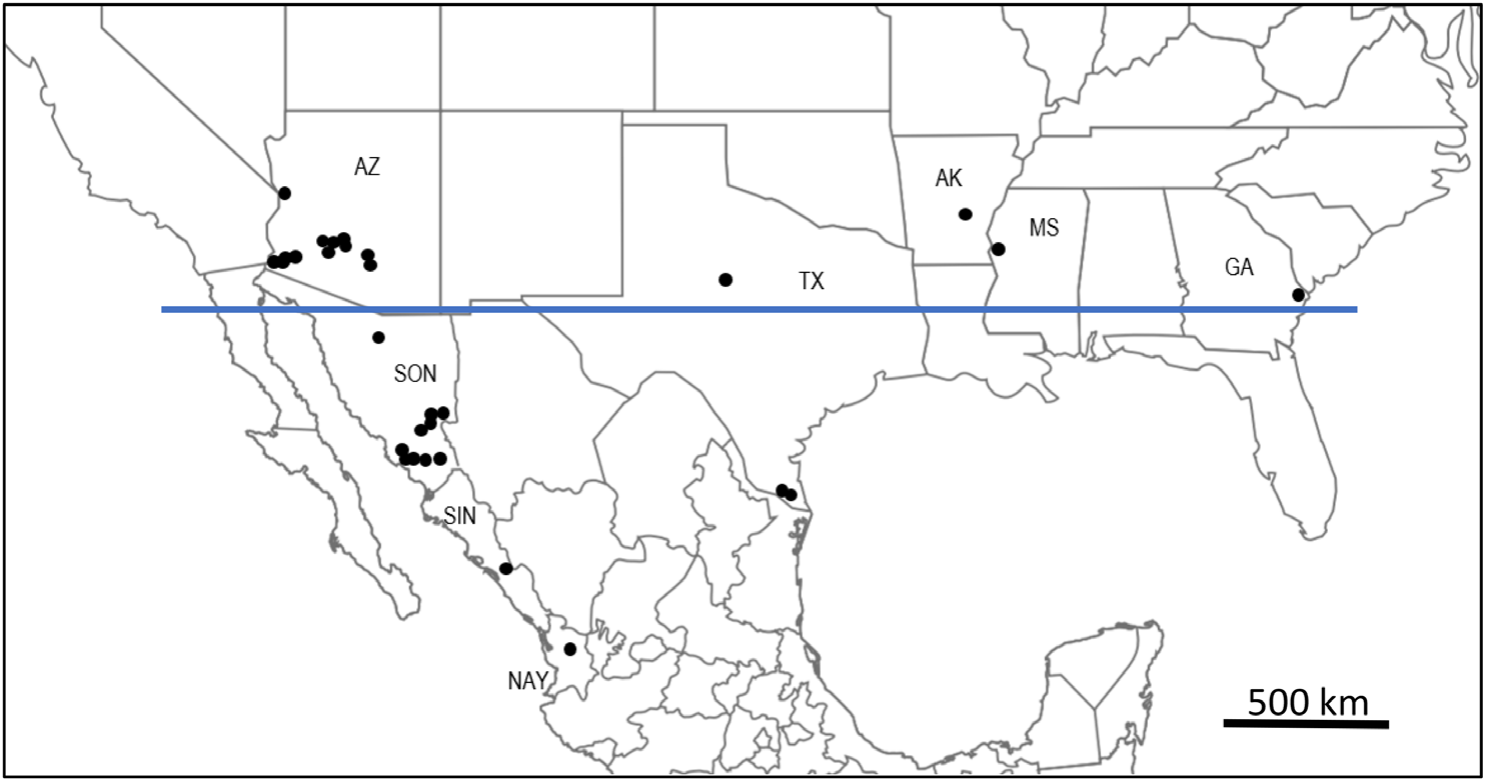
Approximate collection locations of *Aspergillus flavus* VCG YV150 isolates used in the current study. Isolates were recovered from different substrates from 1987 to 2008. AZ, Arizona, USA; TX, Texas, USA; AK, Arkansas, USA; MS, Mississippi, USA; GA, Georgia, USA; SON, Sonora, Mexico; SIN, Sinaloa, Mexico; NAY, Nayarit, Mexico. No isolates with *MAT1‐2* idiomorph were detected below the blue line. [Color figure can be viewed at wileyonlinelibrary.com]

Each of the 82 VCG YV150 isolates (Table 1; Fig. 1) produced a single amplicon (Ramirez‐Prado *et al*., 2008), either a PCR product 395 bp in length associated with *MAT1‐1* or a PCR product 273 bp in length associated with *MAT1‐2* (Table 2). All 48 isolates from Mexico contained the *MAT1‐1* idiomorph. Both *MAT1‐1* and *MAT1‐2* isolates were detected in Arizona and Texas. The three isolates in *a priori* population SO are *MAT1‐2*. The YV150 tester pair was developed from an isolate from Arizona cotton field soil collected in 1987 and is *MAT1‐2*. A tester pair was developed from two maize soil isolates from Sonora collected in 2006, both of them are *MAT1‐1*. All 82 isolates formed heterokaryons (complement the nitrate auxotrophy) with one or both isolates from both tester pairs regardless of the isolate mating‐type. Thus, mating‐type alleles in *A*. *flavus* do not function as vegetative compatibility genes in a manner similar to that reported for *Neurospora crassa* (Perkins, 1988).

**Table 1:** Isolates of *Aspergillus flavus* VCG YV150 used in this study by year of collection, substrate, sample location and *a priori* population.

Year of collection	Substrate	Location	Isolates	*A priori* population^a^
1987	Cottonseed soil	Arizona, USA	1	AZ1
1991	Cottonseed	Arizona, USA	1	AZ1
1992	Cottonseed	Arizona, USA	1	AZ1
1993	Cottonseed	Arizona, USA	1	AZ1
1991	Cottonseed	Arkansas, USA	1	SO
1991	Cottonseed	Georgia, USA	1	SO
1991	Cottonseed	Mississippi, USA	1	SO
1991	Cottonseed	Texas, USA	1	TX
1992	Cottonseed	Texas, USA	1	TX
2000	Cottonseed	Texas, USA	2	TX
2000	Cottonseed	Arizona, USA	6	AZ2
2001	Cottonseed	Arizona, USA	2	AZ2
2002	Cottonseed	Arizona, USA	5	AZ2
2006	Cottonseed soil	Arizona, USA	10	AZ2
2004	Maize	Nayarit, Mexico	1	CC
2006	Maize soil	Sinaloa, Mexico	1	CC
2006	Maize soil	Sonora, Mexico	39	SON1
2007	Maize soil	Sonora, Mexico	3	SON2
2008	Maize soil	Sonora, Mexico	4	SON3

^a^ *A priori* populations were determined by geographic origin and years of isolation. AZ1: Arizona 1987–1993; SO: Southern United States, Georgia, Mississippi, and Alabama from 1991; TX: Texas from 1991, 1992 and 2001; AZ2: Arizona 2000–2006; CC: Central Coast of Mexico, Nayarit 2004 and Sinaloa 2006; SON1: Sonora 2006, SON2: Sonora 2007; SON3: Sonora 2008.

**Table 2:** Frequencies of mating‐type idiomorphs^a^ in *Aspergillus flavus* VCG YV150 *a priori* populations.

*A priori* population^b^	*n* ^c^	*MAT1‐1*	*MAT1‐2*
AZ1	4	0.25	0.75
SO	3	0	1.0
TX	4	0.75	0.25
AZ2	23	0.04	0.96
CC	2	1.0	0
SON1	39	1.0	0
SON2	3	1.0	0
SON3	4	1.0	0

^a^ Mating‐type idiomorphs were identified by the size of amplicons of portions of *MAT1‐1* and *MAT1‐2* (Ramirez‐Prado *et al*., 2008).

^b^ *A priori* populations were determined by geographic origin and year of isolation. AZ1: Arizona 1987–1993; SO: Southern United States, Georgia, Mississippi, and Alabama from 1991; TX: Texas from 1991, 1992 and 2001; AZ2: Arizona 2000–2006; CC: Central Coast of Mexico, Nayarit 2004 and Sinaloa 2006; SON1: Sonora 2006, SON2: Sonora 2007; SON3: Sonora 2008.

^c^ Number of isolates in *a priori* populations.

### Microsatellite genotyping and allelic diversity

For each of the 82 YV150 isolates, 23 loci produced single peaks in the expected range and were relatively easy to score. Locus AF26 was not amplified in any YV150 isolate, regardless of the idiomorph they contained, and was excluded from analyses. Only one locus was monomorphic across all isolates (AF66) (Supplementary Table 1). Over 20% of isolates (20 total) were arbitrarily selected to verify results by re‐amplification. Uncertain alleles were triple‐checked.

### Determination of a posteriori populations

**Fig 2:**
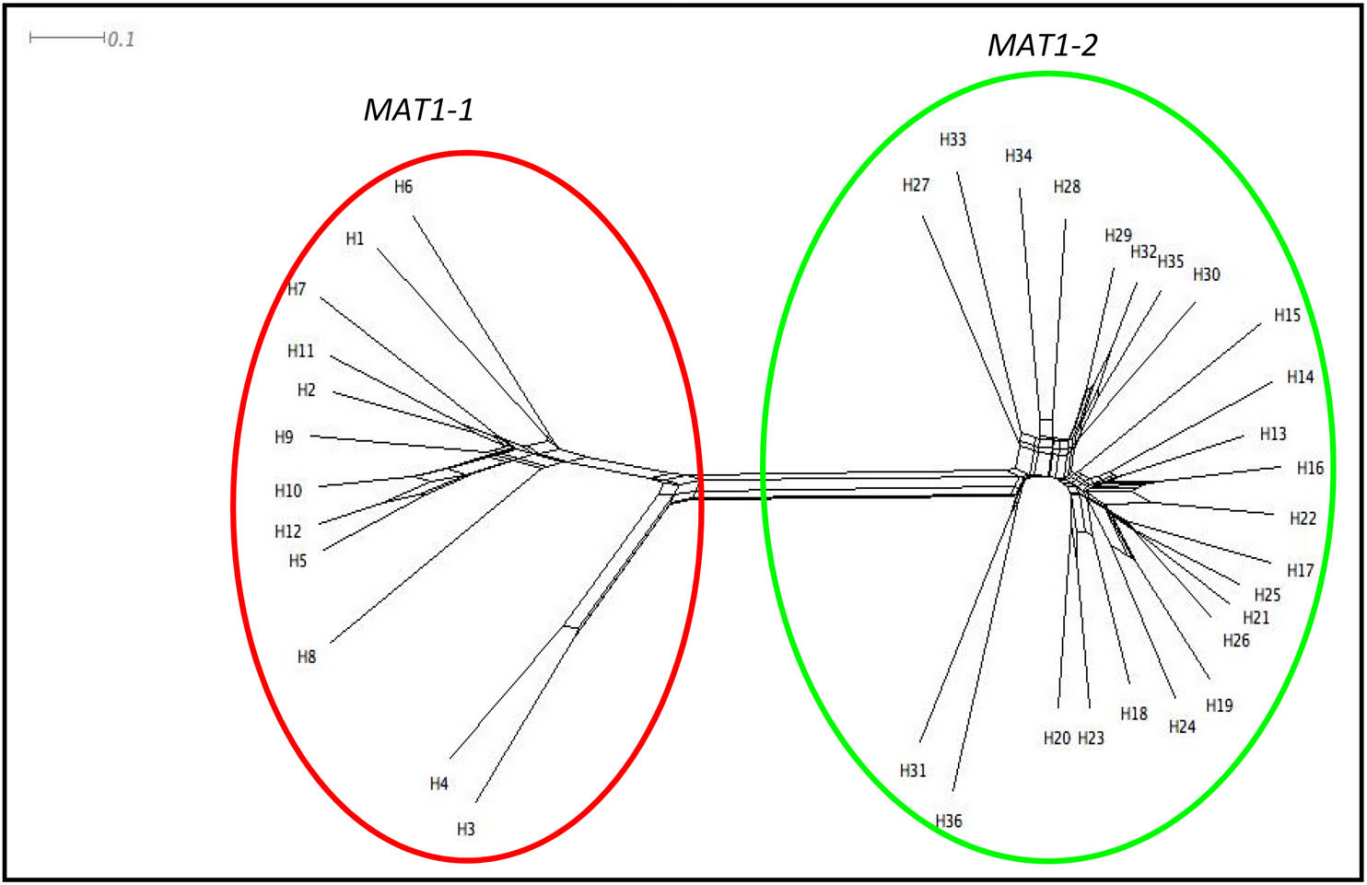
The output of SplitsTree using the neighbour net and phi‐test approaches (Huson and Bryant, 2006). Out of 36 haplotypes, two *a posteriori* populations were determined: *MAT1‐1* and *MAT1‐2* populations. [Color figure can be viewed at wileyonlinelibrary.com]

The network generated in SplitsTree assigned isolates into two clusters (Fig. 2), considered as *a posteriori* populations (Table 3). *MAT1‐2* isolates—all from the United States—composed a single group, while *MAT1‐1* composed another group, regardless of geographical origin and year of isolation. Principal coordinates analysis (PCoA) results also yielded two populations (data not shown) identical to those detected with SplitsTree. In PCoA, the first two axes accounted for 99.48% (94.65% and 4.83%, first and second axis respectively) of the genetic variation. Only 0.25% of the variation was explained by the third axis.

**Table 3:** Descriptive statistics of *Aspergillus flavus* VCG YV150 *a posteriori* populations.

*A posteriori* population^a^	*n* ^b^	*H* ^c^	PL^d^	NA (range)^e^	*D* ^f^	*E* ^g^	$\bar{r}$_d_ (*P*‐value)^h^
*MAT1‐1*	53	12 (10)	14	3.0 (2–6)	0.404	0.138	0.21 (<0.001)
*MAT1‐2*	29	24 (22)	11	4.2 (2–14)	0.973	0.687	0.54 (<0.001)

^a^ *A posteriori* populations as determined using SplitsTree (Huson and Bryant, 2006).

^b^ Number of isolates in the *a posteriori* population.

^c^ Number of haplotypes in the *a posteriori* population. Number in parenthesis indicates haplotypes represented by a single isolate.

^d^ Number of polymorphic loci.

^e^ Mean number of alleles across polymorphic loci. Range of alleles is in parenthesis.

^f^ Genetic diversity according to Nei (1987) as (*n*/(*n* − 1))(1 − *∑Pi* ∑ *Pi* ^2^) (Meirmans and Van Tienderen, 2004).

^g^ Evenness, indicating how evenly genotypes are distributed within a population. If genotypes are evenly distributed, then evenness is 1.

^h^ $\bar{r}$_d_ is the index of association for the standardized number of loci. Significance was determined with 1000 simulations by comparing the observed value of $\bar{r}$_d_ under the null hypothesis of random mating (Agapow and Burt, 2001). The most variable loci in *MAT1‐1* (AF10) and in *MAT1‐2* (AF48 and AF64) were excluded.

### Genotypic diversity

Out of 36 haplotypes detected in YV150, 32 were represented by a single isolate (Table 3). The *MAT1‐1* and *MAT1‐2* populations had 14 and 11 polymorphic loci, respectively. Isolates with *MAT1‐2* had up to 14 alleles in a polymorphic locus compared with six in isolates with *MAT1‐1* (Table 3). Linkage disequilibrium, non‐random association among pairs of loci due to lack of recombination, within clone‐corrected haplotypes of these two populations was highly significant (*P <* 0.001, in both cases; Table 3).

### Diversity indices

All haplotypes were still detected within the YV150 population when isolates originating from Sonora soil 2006 were excluded. Only one haplotype was detected during 2006 across the examined area of Sonora and therefore the Shannon–Wiener diversity index, *H* (Begon *et al*., 1996), was 1.0 for that year (Table 4). That haplotype was detected in other years and locations. In contrast, in the same year, around 1000 km away, each of the 10 YV150 isolates detected in Arizona soil had a distinct haplotype; that population had an *H* value of 0.077, indicating very high diversity. The seven isolates recovered from Sonora soil in 2007 and 2008 were composed of six haplotypes and also had high diversity with an *H* of 0.028 (Table 4).

**Table 4:** Haplotype diversity within various populations of *Aspergillus flavus* YV150 in Mexico and the United States.

YV150 population	*n*	Haplotypes	Haplotype in Sonora soil 2006^a^	Shannon–Wiener diversity index (*H*)	
				Isolates	Haplotype
VCG YV150 minus Sonora soil 2006	43	36	2^b^	0.27	0.0008
Sonora soil 2006	39	1	39	0.23	1.00
Arizona soil 2006	10	10	0	0.01	0.0771
Sonora soil 2007 and 2008	7	6	1^c^	0.01	0.0278

^a^ Number of isolates with a haplotype identical to the haplotype predominant in Sonora soil 2006.

^b^ One in Nayarit, isolate 2006 A/48‐A (although the maize was collected in 2004) and the other in Sonora Soil 2007.

^c^ One in Sonora Soil 2007, see above.

### Population structure

**Fig 3:**
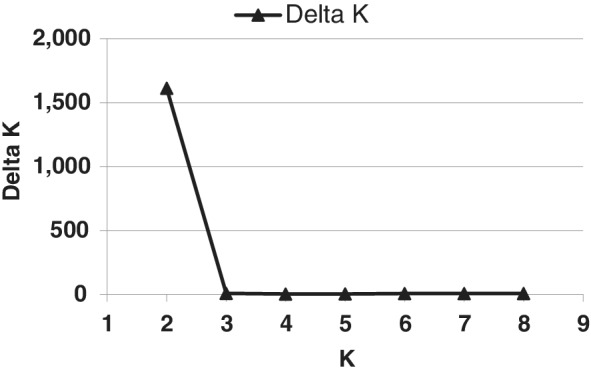
The posterior probability [ln *P*(D)] averaged across 20 simulations for each *K* (data not shown) was used to calculate the optimal number of populations, delta *K*, using Structure Harvester (Earl and VonHoldt, 2012), following the Evanno method (Evanno *et al*., 2005).

**Fig 4:**
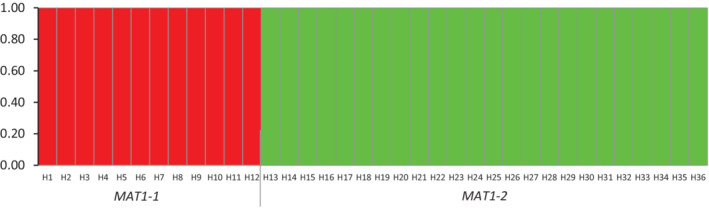
Structure output based on 20 simulations for each *K* (*K* = 1–8, the eight *a priori* populations) for 36 clone‐corrected haplotypes of *Aspergillus flavus* VCG YV150 collected from 1987 to 2008 in the United States and Mexico. The graphic represents the output for one of the simulations for *K* = 2. Each vertical line represents a haplotype along the *x*‐axis. The proportion of membership (*Q*) in a genetic cluster is denoted by colour. Red = alleles associated with *MAT1‐1,* and green = alleles associated with *MAT1‐2*. Haplotypes for isolates containing *MAT1‐1* and *MAT1‐2* are distributed along the *x*‐axis. Haplotypes associated with one mating‐type were not detected in the other. [Color figure can be viewed at wileyonlinelibrary.com]

Assessment of genetic composition was conducted using Structure (Pritchard *et al*., 2000) for clone‐corrected YV150 haplotypes. In the Structure algorithm, the initial conditions were eight *a priori* populations (Table 1). The number of genetic clusters was not clearly indicated with values of the log probability (ln *P*(D)) among 20 successive runs for each *K* (Evanno *et al*., 2005). However, the delta *K* value clearly indicated that the most likely number of genetic clusters (*K*) was two (Δ*K* = 1612) (Fig. 3). Delta *K* was obtained using Structure Harvester (Earl and VonHoldt, 2012), which follows the Evanno method (Evanno *et al*., 2005). There were nine loci in which the same allele occurred in both populations (Supplementary Table 1). However, all *MAT1‐1* haplotypes belong to one cluster and all *MAT1‐2* haplotypes belong to the other (Fig. 4). We did not detect SSR haplotypes associated with both mating types (Fig. 4). Analyses of the partitioning of the genetic variance revealed that 74.5% of the variation was attributed to differences between populations (*P* < 0.0001) and only 25.5% was due to *intra* mating‐type variation (Table 5).

**Table 5:** Analysis of molecular variance for *Aspergillus flavus* VCG YV150 by grouping isolates into two populations, *MAT1‐1* and *MAT1‐*2 populations.

Source of variation	d.f.	Sum of squares	Variance components	% of molecular variation	Fixation indices	*P*‐value^a^
Between *MAT* type	1	172.32	7.12 Va	74.52	FST = 0.7452	<0.0001
Within *MAT* type	46	110.45	2.43 Vb	25.47		
Total	47	282.77	9.55			

^a^ Significance was based on 10 200 permutations.

### Genetic relationships of YV150 with other VCGs with the same range

**Fig 5:**
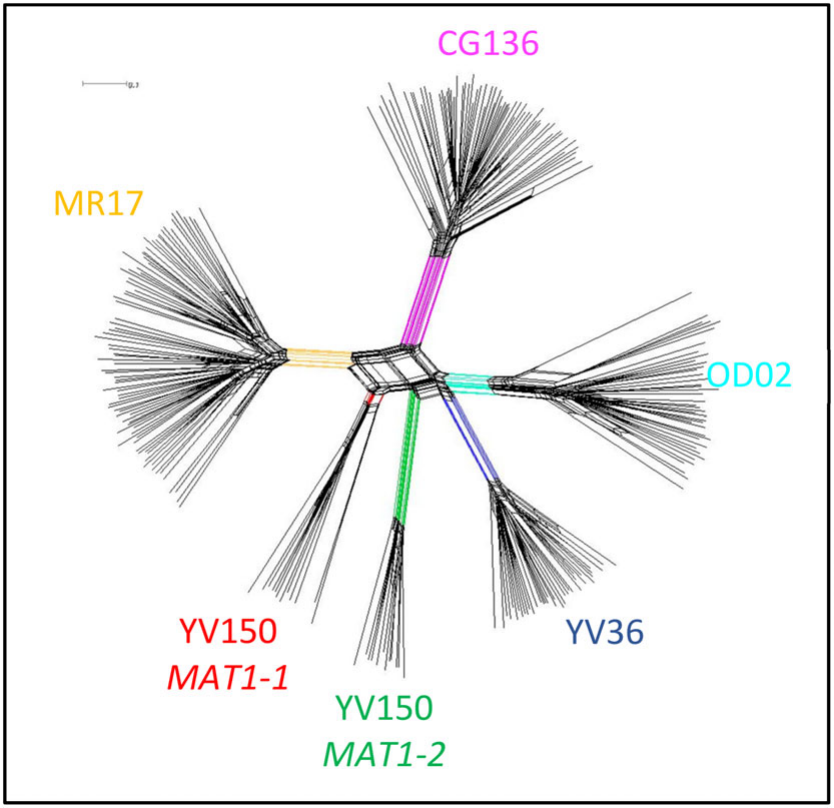
The output of SplitsTree comparing haplotypes of the two YV150 mating‐type idiomorphs with haplotypes of VCGs OD02, MR17, CRG136 (Grubisha and Cotty, 2010) and YV36 (Grubisha and Cotty, 2015) (including the haplotype of AF36, the active ingredient fungus of the aflatoxin biocontrol agent *Aspergillus flavus* AF36). [Color figure can be viewed at wileyonlinelibrary.com]

Members of VCGs OD02, MR17, CRG136, and YV36 have been previously subjected to genetic studies in our laboratory (Grubisha and Cotty, 2010, 2015; Ortega‐Beltran *et al*., 2016). For the five VCG comparison (i.e., OD02, MR17, CRG136, YV36, and YV150), 20 SSR loci were used out of the set of 24 SSR loci (Grubisha and Cotty, 2009). There were four SSR loci that either did not amplify or were highly polymorphic and for that reason were excluded. Locus AF26 did not amplify in any of the isolates of YV150, as mentioned above. Locus AF18 did not amplify in any of the isolates of OD02, as described previously (Grubisha and Cotty, 2010). Loci AF48 and AF64 were highly polymorphic in OD02, as described previously (Grubisha and Cotty, 2010). The second network generated in SplitsTree revealed that members of each VCG and of each of the two mating‐type idiomorphs of YV150 comprised single clusters (Fig. 5).

## Discussion

Populations of the *Aspergillus flavus* L morphotype resident in agricultural fields are mosaics of many VCGs (Bayman and Cotty, 1991; Horn and Greene, 1995; Ortega‐Beltran and Cotty, 2018). SSR haplotypes vary among members of each VCG and linkage equilibrium exists among members of each of the four VCGs previously examined (Grubisha and Cotty, 2010, 2015; Ortega‐Beltran *et al*., 2016). Linkage equilibrium among unlinked SSR loci suggests that genetic exchange within VCGs occurs (Grubisha and Cotty, 2015). SSR loci are not in equilibrium between VCGs, and recombination has not been detected among the five VCGs examined to date (Fig. 5) (Grubisha and Cotty, 2010, 2015; Ortega‐Beltran *et al*., 2016).

Frequencies of individual VCGs within a single field can change several fold between seasons (Bayman and Cotty, 1991), and significant shifts in frequencies of several different VCGs have occurred across an entire region (Ortega‐Beltran and Cotty, 2018). Each shift occurred in a single year, in many maize fields within four agro‐ecosystems distributed over large areas of Sonora, Mexico. The shifts were attributed to founder events, possibly during colonization of organic matter associated with crop development, magnified by the rapid, copious conidiation of the L morphotype and efficient dispersal of the conidia. The largest shift, in terms of the proportion of an *A*. *flavus* population composed of a single VCG, was from 75.5% to 1.7% and occurred between 2006 and 2007 in VCG YV150. However, that study utilized only VCA and different haplotypes in VCG YV150 could not be distinguished. Thus, it was not clear if increased frequencies of YV150 were attributable to a single haplotype, as might be expected from the effects of a founder event, or to an adaptive response shared by all haplotypes within YV150. The results of the current study indicate that the large increase in VCG YV150 involved only a single haplotype containing the *MAT1‐1* idiomorph (Table 4). The single clone detected in all 27 maize fields from across 200 km^2^ in Sonora in 2006 (Ortega‐Beltran and Cotty, 2018) was also detected in one maize soil from Sonora in 2007 and one native maize landrace grain sample from Nayarit in 2004. Founder events such as these may be typical of *A*. *flavus* biology and an aspect of how annually cropped plants become associated with different communities of VCGs over time. Indeed, the trigger of founder events by application of atoxigenic genotypes may be an important component of how biocontrol products based on atoxigenic *A*. *flavus* strains alter *A*. *flavus* communities to reduce the average aflatoxin‐producing ability and thus the extent to which crops become contaminated (Bandyopadhyay *et al*., 2016; Ortega‐Beltran and Cotty, 2018). The presence of a single haplotype in YV150 and the high frequency of YV150 in the *Aspergillus flavus* community as a whole are both consistent with rapid population expansion from a founder event within YV150. While there is no data from 2005 to distinguish an extreme bottleneck due to selection from one due to a founder event, it is usefully modelled as a founder event because of its similarity to what is observed when treating crops with atoxigenic biocontrol strains in which the new population on annual crops has greatly reduced variability from the soil population and primarily reflects the biocontrol haplotype/VCG itself (Bandyopadhyay *et al*., 2016; Ortega‐Beltran and Cotty, 2018).

The use of atoxigenic L morphotype genotypes as active ingredients of biocontrol products causes shifts in *A*. *flavus* population composition, so that the applied genotypes compose most of *A*. *flavus* associated with treated crops (Cotty *et al*., 2007; Bandyopadhyay *et al*., 2016). These induced changes in population structure result in lower crop aflatoxin content. SSR haplotypes of atoxigenic *A*. *flavus* active ingredients are stable over multiple years of biocontrol applications (Grubisha and Cotty, 2015). In both population shifts caused by atoxigenic strain use in agriculture and in the natural event examined in the current work, individual SSR haplotypes dominate the populations after the shift has occurred. However, differences are apparent between the natural founder events described in the current and previous studies (Grubisha and Cotty, 2015; Ortega‐Beltran and Cotty, 2018) and founder events induced by atoxigenic strain biocontrol applications. Atoxigenic strain biocontrol products have residual influence, and atoxigenic strains frequently exceed 90% of the *A*. *flavus* soil population 1 year after treatment and over 30% 3 years after treatment (Cotty, 2000; Cotty *et al*., 2007). In contrast, all the natural population fluctuations discussed here quickly dissipated to background levels by the season following high detection (Ortega‐Beltran and Cotty, 2018). The natural founder events were all detected in the soil. However, natural dispersal over such a large area must involve aerial dispersal of conidia (Brown and Hovmøller, 2002; Isard *et al*., 2005; Aylor, 2018), colonization of above‐ground organic resources, and incorporation into the soil either by cultivation or natural processes. Thus, the population shifts detected in the soil occurred in the spring or summer after the founder event. Failure of the natural population shifts to persist as long as those from an atoxigenic biocontrol application may be attributable to smaller sizes of the *A*. *flavus* communities over much of the examined area, a less‐extreme initial shift, and possibly removal or degradation of crop debris such as stalk and cobbs, which harbour long‐term *A*. *flavus* reservoirs (Ashworth *et al*., 1969; Jaime‐Garcia and Cotty, 2004; Mehl and Cotty, 2010, 2013). Colonized organic matter may have a greater long‐term impact on *A*. *flavus* populations than the ephemeral thin‐walled *A*. *flavus* conidia. The short‐term influence of the founder events described here should caution against discontinuing biocontrol applications even after extensive population modification has been achieved.

In total, 82 isolates of *A*. *flavus* VCG YV150 were examined by SSR characterization in the current study, and 41 of these are the single haplotype that participated in the founder event detected in 2006 Sonoran soil. The remaining 41 YV150 isolates represented 35 SSR haplotypes that had been collected over 20 years across large regions of the United States and Mexico (Table 1). This level of SSR haplotype diversity (i.e., few haplotypes represented by multiple isolates) is common among members of all VCGs examined to date (Grubisha and Cotty, 2010, 2015; Ortega‐Beltran *et al*., 2016). The great diversity of *A*. *flavus* populations is shaped by clonal reproduction and mutation‐driven evolution (Islam *et al*., 2018).

Members of a VCG may be considered to descend from the same clonal lineage and to be more similar to each other than to members of other VCGs (Bayman and Cotty, 1993; Leslie, 1993; Horn and Greene, 1995; Grubisha and Cotty, 2010). However, unlike other VCGs examined in detail by SSR, diversity in YV150 was distributed across isolates containing one or the other of the mating‐type idiomorphs with 35.3% of haplotypes associated with the *MAT1‐1* idiomorph and 64.7% with the *MAT1‐2* idiomorph. There are other aflatoxin‐producing VCGs reported to contain isolates with opposite mating‐type idiomorphs, but in those VCGs only a single member of the less common idiomorph has been identified (Ramirez‐Prado *et al*., 2008; Horn *et al*., 2009b; Sweany *et al*., 2011).

Structure, SplitsTree, and PCoA analyses of SSR data from the current study indicate that the YV150 haplotypes represent two distinct populations, one containing *MAT1‐1* and the other *MAT1‐2* (Figs. 2 and 4). Introgression of haplotypes associated with one mating‐type population into the other was not detected with Structure, suggesting the absence of hybridization between populations (Pritchard *et al*., 2000) despite their co‐occurrence in some areas. Analysis of molecular variance (AMOVA) results also indicate a lack of recombination between isolates with opposite mating‐type (Table 5). The divergence between the *MAT1‐1* and *MAT1‐2* populations (Figs. 2 and 5) is similar to divergences among all four previously examined VCGs (Fig. 5). However, there is no evidence that the separation of the two populations is a result of either characteristics or activities of the mating‐type idiomorphs. From the current results, it is not clear if the distinct structures of the *MAT1‐2* and *MAT1‐1* populations derived through divergence from a common ancestral population with both idiomorphs or from two separate ancestral populations developing vegetative compatibility. Knowledge of the molecular characteristics of *het* loci, which dictates self‐identify within a VCG, is currently insufficient to address this.

Vegetative compatibility has frequently been used to characterize variability in *A*. *flavus* populations (Bayman and Cotty, 1991; Horn and Greene, 1995). As in the present study, most studies determine membership in a VCG with a functional assay that pairs complementary nitrate auxotrophs with a nitrate non‐utilizing (*nit*) mutant from each isolate being evaluated. Complementation of the *nit* mutants indicates membership in the VCG. Previously, four VCGs with many members identified in this manner and collected from sympatric populations distributed over large areas have been examined with an SSR population genetic approach (Grubisha and Cotty, 2010, 2015; Ortega‐Beltran *et al*., 2016). In each case, recombination was not detected between members of different VCGs, but gene flow and allelic equilibrium were detected among individuals within each VCG. Each VCG also contained only a single mating‐type idiomorph. In the current study, VCG YV150 differs from this trend. Although auxotroph complementation puts isolates containing *MAT1‐1* into the same VCG as isolates containing *MAT1‐2*, the population genetic analyses indicate that recombination has not occurred recently between *MAT1‐2* and *MAT1‐1* populations in the field.

The current results suggest that each mating‐type idiomorph of VCG YV150 has a predominantly clonal reproduction mode and that recombination between YV150 isolates with opposite mating‐type is rare, if occurring at all. In predominantly clonally reproducing pathogens, recombination and genetic exchange is not ruled out but is considered too rare to have a significant influence on population clonal structure (Tibayrenc and Ayala, 2012, 2017). If recombination occurred between YV150 isolates with opposite mating‐type idiomorph, there could have been two non‐mutually exclusive scenarios (Grubisha and Cotty, 2010). The first one is that microsatellite loci would be re‐arranged in the resulting progeny. The second is that new arrangements of microsatellite loci would not occur, but recombination would be noticed by detecting infiltration of haplotypes associated with *MAT1‐1* into *MAT1‐2*, or vice versa. Neither of those scenarios was detected in the current study.

Significant research efforts have revealed the sexual cycle of *A*. *flavus* and other aflatoxin‐producing species both in the laboratory and after field release of sclerotia produced and incubated in the laboratory (Horn *et al*., 2009a, 2009b, 2014, 2016; Olarte *et al*., 2012). Highly artificial conditions were necessary to observe low‐frequency recombination events. Thus, the epidemiological significance of *A*. *flavus* recombination in nature remains unknown (Fedorova *et al*., 2009; Kwon‐Chung and Sugui, 2009; Horn *et al*., 2009a, 2011, 2016; Dyer and O'Gorman, 2012). Over a decade ago, it was suggested that if recombination occurred in *A*. *flavus*, such an event would be more likely to occur between isolates belonging to the same VCG (Ehrlich *et al*., 2007; Fedorova *et al*., 2009). Sexual reproduction was not detected in *A*. *parasiticus* when members of a VCG with opposite mating‐type were paired under artificial conditions (Horn *et al*., 2009b). Thus, there are several lines of evidence that indicate sexual recombination is rare in *A*. *flavus* including *in vitro* pairings (Horn *et al*., 2016), observations of undetectable recombination among VCGs (Grubisha and Cotty, 2010) and linkage disequilibria in several populations (Grubisha and Cotty, 2010; Islam *et al*., 2018; Table 3).

The *MAT1‐1* and *MAT1‐2* populations differ in geographic distribution. *MAT1‐2* isolates were not found in Mexico. Factors favouring dominance of *MAT1‐1* isolates in Mexico are unknown, although all were associated with maize production. *MAT1‐1* and *MAT1‐2* are sympatric in both Arizona and Texas (Table 2). *MAT1‐2* was isolated only from cotton fields in the United States. Cropping systems and rotations influence compositions of *A*. *flavus* populations (Horn and Greene, 1995; Jaime‐Garcia and Cotty, 2010) and isolates belonging to different VCGs differ in abilities to colonize diverse substrates (Mehl and Cotty, 2010, 2013). Planting of cotton or maize may thus influence frequencies in the environment of isolates belonging to the two populations of YV150. Around 120 000 ha of cotton are planted annually in Mexico (SIACON, 2010; FAO, 2016), and most of the production occurs in Tamaulipas and Sinaloa. Comparison of *A*. *flavus* communities associated with maize and cottonseed in Tamaulipas and Sinaloa may allow rigorous testing of influences of crops on the prevalence of isolates of the two YV150 populations.

In the current study, clonal reproduction and isolation similar to that seen between VCGs were detected between isolates harbouring different idiomorphs in the same VCG. Genetic isolation exists among sympatric VCGs co‐distributed across large regions of Arizona and Texas (Grubisha and Cotty, 2010) and in VCG YV36 to which the atoxigenic biocontrol agent *Aspergillus flavus* AF36 belongs (Grubisha and Cotty, 2015; Ortega‐Beltran *et al*., 2016) and this is also the case for the *MAT1‐1* and *MAT1‐2* populations of YV150.

## Experimental procedures

*Aspergillus flavus* isolates and assignment into *VCG YV150*. YV150 is one of the first VCGs characterized in our laboratory and has been subjected to several studies, both published and unpublished. After completing the characterization of communities associated with maize in Sonora at the VCG level (Ortega‐Beltran and Cotty, 2018), we investigated whether VCGs from the collection in Arizona (which include VCGs from several areas in the United States) were also present in Sonora. The results of that investigation revealed that YV150 and SON003 are the same VCG. There were over a hundred thousand *A*. *flavus* isolates examined during the period in which the isolates were collected, 1988–2008. Eighty‐two isolates belonging to *A*. *flavus* VCG YV150 from different years, substrates, and geographic origins were used in the current study (Table 1; Fig. 1). Isolates from Sonoran maize field soils collected in 2006, 2007 and 2008 (46 isolates) (Ortega‐Beltran and Cotty, 2018) and one isolate from the United States were previously reported (Ehrlich and Cotty, 2004; Mehl and Cotty, 2010). The remainder of YV150 isolates were collected from crop and soil samples from Mexico (two isolates) and the United States (33 isolates) using previously described methods (Cotty, 1997; Probst *et al*., 2010). Soil and maize samples from Mexico were imported into the United States under an APHIS *Permit to Move Live Plant Pests and Noxious Weeds*, USDA‐APHIS‐PPQ 526, and maintained at the USDA‐ARS Aflatoxin Research Laboratory in the School of Plant Sciences, University of Arizona, Tucson. A single isolate from maize kernels was obtained from a native maize landrace accession collected in 2004. Isolates from Mexico, from both maize and maize field soils, and from Arizona cotton field soils from 2006 were recovered by dilution plate technique on modified rose Bengal agar (Cotty, 1994). Cottonseed isolates from 1991 through 1993 were obtained by surface‐disinfecting seeds with 95% ethanol and plating directly into 5‐2 agar (Cotty, 1989). The assignment of isolates into VCG YV150 was conducted using *nit* mutants in VCA on complementation agar (Cotty and Taylor, 2003) following previously described protocols (Ortega‐Beltran and Cotty, 2018). In 2006, a total of 27 maize fields within four agro‐ecosystems encompassing 200 km^2^ and at elevations ranging from 6 to over 2100 m above sea level were sampled, and YV150 was detected in each maize field (Ortega‐Beltran and Cotty, 2018). From 26 fields, we arbitrarily selected one YV150 isolate. From the remaining field, we selected all 11 YV150 detected in that field. All evaluated YV150 isolates produce aflatoxins.

### Microsatellite genotyping

Isolates were genotyped using 24 microsatellite markers for *A*. *flavus* (Grubisha and Cotty, 2009). DNA isolation, multiplex‐PCR, and microsatellite genotyping were conducted as previously described (Grubisha and Cotty, 2009, 2010; Callicott and Cotty, 2015). In order to assess the consistency of the data, over 20% of isolates were subjected to at least three independent PCR and genotyping assays for all loci.

### Mating‐type idiomorph characterization

Mating‐type idiomorphs of VCG YV150 isolates were characterized by multiplex‐PCR amplification of segments of *MAT1‐1* and *MAT1‐2* using primers M1F, M1R, M2F, and M2R (Ramirez‐Prado *et al*., 2008) with minor modifications (Grubisha and Cotty, 2010).

### Population genetic analyses

Isolates were placed into eight *a priori* populations based on year of isolation and geographic origin (Table 1). Haplotypes, allele frequencies and evenness among isolates with *MAT1‐1* and *MAT1‐2* were assessed independently with GENODIVE 2.0b11 (Meirmans and Van Tienderen, 2004). Evenness indicates haplotype distribution within each population.

A Cavalli‐Sforza chord distance matrix obtained with GENODIVE was used to generate a phylogenetic tree using SplitsTree 4.8 (Huson and Bryant, 2006). Recombination and genetic distance among YV150 haplotypes were evaluated with the neighbour‐net approach and the phi‐test of SplitsTree. This approach uses a jackknife strategy and repeats the phi‐test after each individual is removed and subsequently replaced. *A posteriori* populations were then determined. The covariance matrix of the Φ_PT_ distance matrix was used to perform a PCoA in GENALEX (Peakall and Smouse, 2006) and *a posteriori* populations were confirmed.

Partitioning of genetic variance was calculated considering isolates with *MAT1‐1* as one population and isolates with *MAT1‐2* as a different population with a hierarchical AMOVA using Arlequin 3.5 (Excoffier and Lishcer, 2010). Significance was based on 10 200 permutations. Loci AF64 and AF48 were removed from the *MAT1‐2* population because of allelic variability (eight and 14 alleles respectively). Increased variability may be the result of loci under selection (Grubisha and Cotty, 2010). No loci were removed for the *MAT1‐1* population since the maximum number of alleles in any given locus was six.

Linkage disequilibrium between pairs of polymorphic loci of *a posteriori* populations was estimated using MULTILOCUS 1.3b (Agapow and Burt, 2001). Statistical significance was determined by 1000 permutations. The most variable loci within each *a posteriori* population were excluded. Linkage disequilibrium was estimated using the index of association ($\bar{r}$_d_) standardized for the included loci. Significance was based on 1000 randomizations and compared the observed value of $\bar{r}$_d_ to that of expected under the null hypothesis of random mating (Agapow and Burt, 2001).

Genetic structure was assessed with Bayesian clustering program Structure 2.2.3 (Pritchard *et al*., 2000) to define genetic groups within VCG YV150. Haplotypes were assigned to *K* populations using the admixture model and default parameters. Markov Chain Monte Carlo (MCMC) simulations were run for *K* = 1–8 (eight *a priori* populations; Table 1). Simulations (20 total) were run with a burn‐in length of 100 000 MCMC generations followed by 1 million MCMC iterations for each *K*. The optimal number of populations was obtained using Structure Harvester (Earl and VonHoldt, 2012), which calculates the rate of change in the log probability of data between successive runs of *K* (Evanno *et al*., 2005).

A second phylogenetic tree was generated by assessing recombination and genetic distance between YV150 haplotypes and haplotypes of VCGs OD02, MR17, CRG136, (Grubisha and Cotty, 2010) and YV36 (Grubisha and Cotty, 2015) (including the haplotype of AF36, the active ingredient fungus of the aflatoxin biocontrol product *Aspergillus flavus* AF36 Prevail®). A Cavalli‐Sforza chord distance matrix obtained with GENODIVE was used to generate a phylogenetic tree using SplitsTree as above.

### Diversity indices

Shannon–Wiener diversity index (*H*) was calculated for both haplotypes and isolates of diverse YV150 populations as follows: (i) all except those found in Sonora soil 2006, (ii) those found in Sonora soil 2006, (iii) those found in Arizona soil 2006, and (iv) those found in Sonora soil 2007 and Sonora soil 2008. The formula used was $H=-\sum_{i=1}^{S}\mathrm{Pi}\times \ln\mathrm{Pi}$ where *Pi* is the proportion for the *i*th haplotype and *S* is the total number of haplotypes in YV150 detected in the current study (Begon *et al*., 1996). The same formula was used to calculate *H* for isolates but in this case, *Pi* is the proportion for the *i*th isolate and *S* is the total number of isolates of YV150 used in the current study.

## Supporting information


**Supplementary Table 1** Number of alleles per locus found in the examined *MAT1‐1* and *MAT1‐2* populations. The last column (*MAT1‐1* & *MAT1‐2*) indicates the number of alleles in a given locus occurring in both populations.

**Appendix** **S1:** Supporting information
