# Supplementary material for: Founder events influence structures of Aspergillus flavus populations
Source: Environ Microbiol. 2020 Jun 27;22(8):3522–34. doi: 10.1111/1462-2920.15122 (PMC7496522; doi:10.1111/1462-2920.15122)
Supplement: Supplementary file 1 — Supplementary Table 1 Number of alleles per locus found in the examined MAT1‐1 and MAT1‐2 populations. The last column (MAT1‐1 & MAT1‐2) indicates the number of alleles in a given locus occurring in both populations. [file EMI-22-3522-s002.docx]

**Supplementary Table 1** Number of alleles per locus found in the examined *MAT1-1* and *MAT1-2* populations. The last column (*MAT1-1* & *MAT1-2*) indicates the number of alleles in a given locus occurring in both populations.

| Locus | *MAT1-1* | *MAT1-2* | *MAT1-1 & MAT1-2* |
| --- | --- | --- | --- |
| AF18 | 2 | 3 | 0 |
| AF16 | 2 | 2 | 0 |
| AF28 | 2 | 1 | 0 |
| AF22 | 2 | 1 | 0 |
| AF33 | 2 | 1 | 0 |
| AF27 | 2 | 1 | 0 |
| AF17 | 2 | 1 | 0 |
| AF42 | 3 | 2 | 0 |
| AF34 | 3 | 1 | 0 |
| AF63 | 3 | 1 | 0 |
| AF54 | 4 | 1 | 0 |
| AF64 | 5 | 7 | 0 |
| AF31 | 5 | 1 | 0 |
| AF8 | 6 | 2 | 0 |
| AF66 | 0 | 0 | 1 |
| AF55 | 1 | 0 | 1 |
| AF43 | 2 | 1 | 1 |
| AF53 | 2 | 1 | 1 |
| AF11 | 2 | 1 | 1 |
| AF25 | 3 | 0 | 1 |
| AF48 | 4 | 16 | 1 |
| AF10 | 10 | 1 | 1 |
| AF13 | 2 | 2 | 2 |
