# Supplementary material for: Founder events influence structures of Aspergillus flavus populations
Source: Environ Microbiol. 2020 Jun 27;22(8):3522–34. doi: 10.1111/1462-2920.15122 (PMC7496522; doi:10.1111/1462-2920.15122)
Supplement: Supplementary file 2 — Appendix S1: Supporting information [file EMI-22-3522-s001.docx]

**Supplementary Table for Review 1**. Information of the 82 *Aspergillus flavus* isolates belonging to vegetative compatibility group (VCG) YV150 used in the current study: isolate name, year of isolation, state of origin in the US or Mexico, coordinates and elevation of sample location site, mating-type idiomorph (Ramirez-Prado et al. 2008), clone number based on alleles calls (Supplementary Table for Review 2), and *a priori* population based on year and location of isolation.

| **Isolate name** | **Isolate code** | **Year of isolation** | **State** | **N** | **W** | **masl** | **MAT** | **Clone** | ***A priori* population** |
| --- | --- | --- | --- | --- | --- | --- | --- | --- | --- |
| 2006 A/48 A | C27 | 2006 | Nayarit | 22° 01' 11.5" | 104° 52' 32.6" | 457 | 1-1 | 1 | Southern Mexico |
| SSS'06 MN-E | Son8 | 2006 | Sonora | 27° 03' 55.0" | 109° 00' 21.1" | 490 | 1-1 | 1 | Sonora 2006 |
| SSS'06 EP2-G | Son10 | 2006 | Sonora | 27° 59' 59.3" | 109° 18' 42.9" | 648 | 1-1 | 1 | Sonora 2006 |
| SSS'06 A1-N | Son11 | 2006 | Sonora | 27° 01' 28.1" | 108° 55' 11.2" | 371 | 1-1 | 1 | Sonora 2006 |
| SSS'06 Ya8-D | Son12 | 2006 | Sonora | 26° 58' 41.1" | 109° 33' 35.8" | 20 | 1-1 | 1 | Sonora 2006 |
| SSS'06 A4-E | Son19 | 2006 | Sonora | 27° 04' 30.3" | 109° 03' 53.9" | 369 | 1-1 | 1 | Sonora 2006 |
| SSS'06 T2-L | Son26 | 2006 | Sonora | 27° 50' 30.1" | 109° 22' 40.7" | 442 | 1-1 | 1 | Sonora 2006 |
| SSS'06 C2-J | Son29 | 2006 | Sonora | 28° 20' 33.8" | 109° 01' 19.9" | 2,116 | 1-1 | 1 | Sonora 2006 |
| SSS'06 Ye5-J | Son30 | 2006 | Sonora | 28° 21' 22.3" | 108° 55' 45.1" | 1,542 | 1-1 | 1 | Sonora 2006 |
| SSS'06 EjES-M | Son32 | 2006 | Sonora | 27° 47' 36.9" | 109° 30' 20.5" | 486 | 1-1 | 1 | Sonora 2006 |
| SSS'06 C3-M | Son36 | 2006 | Sonora | 28° 20' 06.1" | 109° 01' 06.2" | 2,109 | 1-1 | 1 | Sonora 2006 |
| SSS'06 Ye2-A | Son37 | 2006 | Sonora | 28° 23' 50.7" | 108° 55' 16.9" | 1,527 | 1-1 | 1 | Sonora 2006 |
| SSS'06 C1-A | Son38 | 2006 | Sonora | 28° 20' 39.4" | 109° 01' 47.9" | 2,123 | 1-1 | 1 | Sonora 2006 |
| SSS'06 Nuri-C | Son39 | 2006 | Sonora | 28° 07' 42.5" | 109° 19' 17.3" | 348 | 1-1 | 1 | Sonora 2006 |
| SSS'06 EP1-N | Son40 | 2006 | Sonora | 28° 12' 17.6" | 109° 19' 13.9" | 389 | 1-1 | 1 | Sonora 2006 |
| SSS'06 T1-G | Son41 | 2006 | Sonora | 27° 56' 36.6" | 109° 19' 22.9" | 511 | 1-1 | 1 | Sonora 2006 |
| SSS'06 Ya2-F | Son42 | 2006 | Sonora | 27° 30' 22.8" | 110° 03' 22.1" | 30 | 1-1 | 1 | Sonora 2006 |
| SSS'06 Ya5-B | Son43 | 2006 | Sonora | 27° 09' 31.1" | 109° 51' 5.5" | 24 | 1-1 | 1 | Sonora 2006 |
| SSS'06 Ye1-B | Son44 | 2006 | Sonora | 28° 24' 45.1" | 108° 55' 28.4" | 1,583 | 1-1 | 1 | Sonora 2006 |
| SSS'06 Ya7-C | Son45 | 2006 | Sonora | 26° 59' 21.4" | 109° 40' 17.7" | 20 | 1-1 | 1 | Sonora 2006 |
| SSS'06 Ya1-B | Son46 | 2006 | Sonora | 27° 31' 11.3" | 110° 02' 03.8" | 30 | 1-1 | 1 | Sonora 2006 |
| SSS'06 A3-B | Son47 | 2006 | Sonora | 27° 02' 34.6" | 108° 58' 28.3" | 433 | 1-1 | 1 | Sonora 2006 |
| SSS'06 Ya3-G | Son48 | 2006 | Sonora | 27° 25' 02.7" | 110° 05' 49.4" | 33 | 1-1 | 1 | Sonora 2006 |
| SSS'06 Ya6-D | Son49 | 2006 | Sonora | 27° 03' 04.7" | 109° 51' 02.5" | 6 | 1-1 | 1 | Sonora 2006 |
| SSS'06 Ye3-A | Son50 | 2006 | Sonora | 28° 20' 53.4" | 108° 55' 54.8" | 1,561 | 1-1 | 1 | Sonora 2006 |
| SSS'06 NA-I | Son51 | 2006 | Sonora | 27° 04' 34.2" | 109° 18' 27.1" | 102 | 1-1 | 1 | Sonora 2006 |
| SSS'06 Ye4-E | Son52 | 2006 | Sonora | 28° 20' 37.2" | 108° 56' 06.9" | 1,557 | 1-1 | 1 | Sonora 2006 |
| SSS'06 SON003 T1 | Son53 | 2006 | Sonora | 27° 03' 55.0" | 109° 00' 21.1" | 490 | 1-1 | 1 | Sonora 2006 |
| SSS'06 SON003 T2 | Son54 | 2006 | Sonora | 27° 04' 34.2" | 109° 18' 27.1" | 102 | 1-1 | 1 | Sonora 2006 |
| SSS'06 A2-B | Son55 | 2006 | Sonora | 27° 02' 12.4" | 108° 55' 41.1" | 417 | 1-1 | 1 | Sonora 2006 |
| SSS'06 A2-C | Son56 | 2006 | Sonora | 27° 02' 12.4" | 108° 55' 41.1" | 417 | 1-1 | 1 | Sonora 2006 |
| SSS'06 A2-D | Son57 | 2006 | Sonora | 27° 02' 12.4" | 108° 55' 41.1" | 417 | 1-1 | 1 | Sonora 2006 |
| SSS'06 A2-E | Son58 | 2006 | Sonora | 27° 02' 12.4" | 108° 55' 41.1" | 417 | 1-1 | 1 | Sonora 2006 |
| SSS'06 A2-H | Son59 | 2006 | Sonora | 27° 02' 12.4" | 108° 55' 41.1" | 417 | 1-1 | 1 | Sonora 2006 |
| SSS'06 A2-I | Son60 | 2006 | Sonora | 27° 02' 12.4" | 108° 55' 41.1" | 417 | 1-1 | 1 | Sonora 2006 |
| SSS'06 A2-J | Son61 | 2006 | Sonora | 27° 02' 12.4" | 108° 55' 41.1" | 417 | 1-1 | 1 | Sonora 2006 |
| SSS'06 A2-K | Son62 | 2006 | Sonora | 27° 02' 12.4" | 108° 55' 41.1" | 417 | 1-1 | 1 | Sonora 2006 |
| SSS'06 A2-L | Son63 | 2006 | Sonora | 27° 02' 12.4" | 108° 55' 41.1" | 417 | 1-1 | 1 | Sonora 2006 |
| SSS'06 A2-M | Son64 | 2006 | Sonora | 27° 02' 12.4" | 108° 55' 41.1" | 417 | 1-1 | 1 | Sonora 2006 |
| SSS'06 A2-N | Son65 | 2006 | Sonora | 27° 02' 12.4" | 108° 55' 41.1" | 417 | 1-1 | 1 | Sonora 2006 |
| SSS'07 VY3-C | Son66 | 2006 | Sonora | 27° 25' 02.7" | 110° 05' 49.4" | 33 | 1-1 | 1 | Sonora 2007 |
| AF150 | C34 | 1987 | Arizona | 32° 41' 31.6" | 114° 08' 32.3" | 68 | 1-2 | 2 | Arizona 87-93 |
| Az Ext 00 300-B-D | C23 | 2000 | Arizona | 33° 07' 57.7" | 112° 42' 33.6" | 213 | 1-2 | 2 | Arizona 00-06 |
| Maricopa 1210-A-N AZ2 000^a^ | LG280 | 2000 | Arizona |  |  |  | 1-2 | 2 | Arizona 00-06 |
| 91013-E | C14 | 1991 | Arkansas | 34° 13' 41.8" | 92° 00' 10.9" | 67 | 1-2 | 2 | Southern US |
| 91116-D | C10 | 1991 | Georgia | 31° 56' 25.6" | 81°07' 18.6" | 4 | 1-2 | 2 | Southern US |
| Az Ext 02 32115-1012-A-B | C2 | 2002 | Arizona | 32° 45' 13.9" | 114° 30' 10.9" | 46 | 1-2 | 3 | Arizona 00-06 |
| Az Ext 02 32210-2701-A | C3 | 2002 | Arizona | 32° 47' 22.9" | 113° 53' 55.9" | 88 | 1-2 | 3 | Arizona 00-06 |
| SSS'08 EP2-L | C29 | 2008 | Sonora | 28° 07' 18.1" | 109° 11' 29.4" | 648 | 1-1 | 4 | Sonora 2008 |
| SSS'08 EP2-A | C30 | 2008 | Sonora | 28° 07' 18.1" | 109° 11' 29.4" | 648 | 1-1 | 4 | Sonora 2008 |
| 91065-B | C13 | 1991 | Arizona | 32° 55' 16.7" | 111° 55' 35.4" | 394 | 1-2 | 5 | Arizona 87-93 |
| 93072-A | C15 | 1993 | Arizona | 32° 55' 16.7" | 111° 55' 35.4" | 394 | 1-2 | 6 | Arizona 87-93 |
| 91108-C | C12 | 1991 | Mississippi | 33° 24' 18.8" | 90° 53' 50.8 | 38 | 1-2 | 7 | Southern US |
| 92143-E | C16 | 1992 | Texas | 32° 28' 15.1" | 100° 24' 19.8" | 657 | 1-2 | 8 | Texas |
| Az Ext 00 2813-A-K | C20 | 2000 | Arizona | 33° 24' 09.6" | 112° 22' 14.3" | 281 | 1-2 | 9 | Arizona 00-06 |
| Az Ext 00 1011,1008-1010-A-J | C7 | 2000 | Arizona | 32° 44' 52.4" | 114° 00' 02.9" | 78 | 1-2 | 10 | Arizona 00-06 |
| Az Ext 00 1810,1805-A-L | C8 | 2000 | Arizona | 33° 25' 34.3" | 112° 11' 27.5" | 313 | 1-2 | 11 | Arizona 00-06 |
| Az Ext 00 1802-A-F | C9 | 2000 | Arizona | 32° 45' 11.8" | 111° 33' 49.2" | 475 | 1-2 | 12 | Arizona 00-06 |
| Az Ext 01 32207-1504-H | C22 | 2001 | Arizona | 32° 44' 03.3" | 113° 59' 39.1" | 79 | 1-2 | 13 | Arizona 00-06 |
| Az Ext 02 32209-309-5 1/2-B-D | C4 | 2002 | Arizona | 32° 45' 13.7" | 113° 53' 10.1" | 86 | 1-2 | 14 | Arizona 00-06 |
| Az Ext 02 32208-3602-3609-J | C5 | 2002 | Arizona | 32° 46' 06.7" | 113° 57' 48.4" | 83 | 1-2 | 15 | Arizona 00-06 |
| Az Ext 02 31521-103-111-G | C6 | 2002 | Arizona | 33° 01' 28.4" | 112° 06' 29.7" | 374 | 1-2 | 16 | Arizona 00-06 |
| AZSO06 K19-F | N1 | 2006 | SON003 | 33° 22' 33.5" | 112° 38' 45.5" | 274 | 1-2 | 17 | Arizona 00-06 |
| AZSO06 K26-E | N2 | 2006 | SON003 | 33° 23' 26.0" | 112° 38' 49.4" | 287 | 1-2 | 18 | Arizona 00-06 |
| AZSO06 J13-C | N3 | 2006 | SON003 | 33° 24' 17.1" | 112° 29' 29.7" | 281 | 1-2 | 19 | Arizona 00-06 |
| AZSO06 B50-O | N4 | 2006 | SON003 | 32° 47' 38.2" | 113° 49' 50.7" | 91 | 1-2 | 20 | Arizona 00-06 |
| AZSO06 I12-E | N5 | 2006 | SON003 | 32° 52' 37.5" | 111° 55' 50.6" | 399 | 1-2 | 21 | Arizona 00-06 |
| AZSO06 O3-M | N6 | 2006 | SON003 | 33° 26' 18.1" | 112° 14' 34.4" | 309 | 1-2 | 22 | Arizona 00-06 |
| AZSO06 K7-J | N7 | 2006 | SON003 | 33° 24' 09.9" | 112° 42' 23.8" | 295 | 1-2 | 23 | Arizona 00-06 |
| AZSO06 B53-D | N8 | 2006 | SON003 | 32° 43' 00.6" | 114° 05' 32.9" | 70 | 1-1 | 24 | Arizona 00-06 |
| AZSO06 A18-K | N10 | 2006 | SON003 | 32° 51' 59.5" | 111° 39' 23.7" | 441 | 1-2 | 25 | Arizona 00-06 |
| AZSO06 G7-O | N11 | 2006 | SON003 | 34° 54' 12.9" | 114° 34' 24.6" | 143 | 1-2 | 26 | Arizona 00-06 |
| Az Ext 01 31006-3609-4-B | C19 | 2001 | Arizona | 32° 35' 28.2" | 111° 35' 22.8" | 501 | 1-1 | 27 | Arizona 00-06 |
| 91070-B | C18 | 1991 | Texas | 26° 11' 28.9" | 97° 41' 46.6" | 13 | 1-1 | 28 | Texas |
| 92087-F | C11 | 1992 | Arizona | 32° 55' 16.7" | 111° 55' 35.4" | 394 | 1-1 | 29 | Arizona 87-93 |
| NCB-S 19-E | C24 | 2006 | Sinaloa | 24° 04' 43.0" | 106° 22' 14.0" | 230 | 1-1 | 30 | Southern Mexico |
| SSS'07 NA2-O | C31 | 2007 | Sonora | 27° 38' 36.3" | 109° 10' 12.9" | 121 | 1-1 | 31 | Sonora 2007 |
| SSS'07 EP2-A | Son67 | 2007 | Sonora | 27° 59' 59.3" | 109° 18' 42.9" | 648 | 1-1 | 32 | Sonora 2007 |
| SSS'08 EP2-H | C26 | 2008 | Sonora | 28° 07' 18.1" | 109° 11' 29.4" | 648 | 1-1 | 33 | Sonora 2008 |
| SSS'08 EjES-H | C28 | 2000 | Sonora | 27° 28' 25.4" | 109° 18' 7.04" | 475 | 1-1 | 34 | Sonora 2008 |
| ST Ext 00 Progreso-I | C21 | 2000 | Texas | 26° 05' 32.7" | 97° 57' 25.9" | 21 | 1-1 | 35 | Texas |
| RGV Hargill-I TX2000^a^ | LG284 | 2000 | Texas |  |  |  | 1-1 | 36 | Texas |

^a^ Coordinates and elevation of sample collection site were not taken.

**Supplementary Table for Review 2.** Allele sizes of 23 SSR loci (Grubisha and Cotty, 2009) for 82 *Aspergillus flavus* isolates belonging to VCG YV150.

| **Name** | **MAT** | **ID** | **AF28** | **AF13** | **AF43** | **AF22** | **AF31** | **AF42** | **AF18** | **AF48** | **AF8** | **AF53** | **AF10** | **AF33** | **AF34** | **AF27** | **AF16** | **AF54** | **AF17** | **AF11** | **AF66** | **AF64** | **AF63** | **AF25** | **AF55** |
| --- | --- | --- | --- | --- | --- | --- | --- | --- | --- | --- | --- | --- | --- | --- | --- | --- | --- | --- | --- | --- | --- | --- | --- | --- | --- |
| AF150 | 1-2 | C34 | 135 | 161 | 387 | 144 | 308 | 162 | 206 | 482 | 186 | 134 | 285 | 168 | 304 | 104 | 191 | 168 | 364 | 138 | 269 | 209 | 126 | 323 | 174 |
| 91116-D | 1-2 | C10 | 135 | 161 | 387 | 144 | 308 | 162 | 206 | 485 | 186 | 134 | 285 | 168 | 304 | 104 | 191 | 168 | 364 | 138 | 269 | 209 | 126 | 323 | 174 |
| 91108-C | 1-2 | C12 | 135 | 161 | 387 | 144 | 308 | 162 | 206 | 458 | 188 | 134 | 285 | 168 | 304 | 104 | 191 | 168 | 364 | 138 | 269 | 209 | 126 | 323 | 174 |
| 91065-B | 1-2 | C13 | 135 | 161 | 390 | 144 | 308 | 162 | 206 | 488 | 186 | 134 | 285 | 168 | 304 | 104 | 191 | 168 | 364 | 138 | 269 | 209 | 126 | 323 | 174 |
| 91013-E | 1-2 | C14 | 135 | 161 | 387 | 144 | 308 | 162 | 206 | 450 | 186 | 134 | 285 | 168 | 304 | 104 | 191 | 168 | 364 | 138 | 269 | 209 | 126 | 323 | 174 |
| 91070-B | 1-1 | C18 | 119 | 161 | 387 | 183 | 355 | 154 | 147 | 403 | 209 | 144 | 372 | 171 | 300 | 262 | 175 | 176 | 350 | 144 | 269 | 176 | 135 | 333 | 174 |
| 92087-F | 1-1 | C11 | 119 | 161 | 387 | 183 | 361 | 154 | 147 | 412 | 206 | 144 | 383 | 171 | 300 | 262 | 175 | 180 | 350 | 144 | 269 | 178 | 135 | 329 | 174 |
| 92143-E | 1-2 | C16 | 135 | 157 | 387 | 144 | 308 | 162 | 206 | 0 | 186 | 134 | 285 | 168 | 304 | 104 | 191 | 168 | 364 | 138 | 269 | 211 | 126 | 323 | 174 |
| 93072-A | 1-2 | C15 | 135 | 167 | 387 | 144 | 308 | 162 | 213 | 474 | 186 | 134 | 285 | 168 | 304 | 104 | 191 | 168 | 364 | 138 | 269 | 209 | 126 | 323 | 174 |
| Az Ext 00 2813-A-K | 1-2 | C20 | 135 | 161 | 387 | 144 | 308 | 162 | 206 | 447 | 186 | 134 | 285 | 168 | 304 | 104 | 191 | 168 | 364 | 138 | 269 | 213 | 126 | 323 | 174 |
| Az Ext 00 1011,1008-1010-A-J | 1-2 | C7 | 135 | 161 | 387 | 144 | 308 | 165 | 206 | 452 | 186 | 134 | 285 | 168 | 304 | 104 | 191 | 168 | 364 | 138 | 269 | 211 | 126 | 323 | 174 |
| Az Ext 00 1810,1805-A-L | 1-2 | C8 | 135 | 161 | 387 | 144 | 308 | 162 | 209 | 444 | 186 | 134 | 285 | 168 | 304 | 104 | 191 | 168 | 364 | 138 | 269 | 211 | 126 | 323 | 174 |
| Az Ext 00 1802-A-F | 1-2 | C9 | 135 | 161 | 387 | 144 | 308 | 162 | 206 | 432 | 186 | 134 | 285 | 168 | 304 | 104 | 191 | 168 | 364 | 138 | 269 | 211 | 126 | 323 | 174 |
| Az Ext 00 300-B-D | 1-2 | C23 | 135 | 161 | 387 | 144 | 308 | 162 | 206 | 438 | 186 | 134 | 285 | 168 | 304 | 104 | 191 | 168 | 364 | 138 | 269 | 209 | 126 | 323 | 174 |
| Maricopa 1210-A-N AZ2000 | 1-2 | LG280 | 135 | 161 | 387 | 144 | 308 | 162 | 206 | 455 | 186 | 134 | 285 | 168 | 304 | 104 | 191 | 168 | 364 | 138 | 269 | 209 | 126 | 323 | 174 |
| ST Ext 00 Progreso-I | 1-1 | C21 | 125 | 182 | 387 | 183 | 358 | 154 | 147 | 403 | 177 | 144 | 288 | 171 | 300 | 274 | 175 | 165 | 350 | 138 | 269 | 178 | 131 | 323 | 174 |
| RGV Hargill-I TX2000 | 1-1 | LG284 | 125 | 182 | 387 | 183 | 358 | 154 | 147 | 403 | 177 | 144 | 380 | 171 | 300 | 262 | 175 | 165 | 350 | 138 | 269 | 178 | 135 | 323 | 174 |
| Az Ext 01 31006-3609-4-B | 1-1 | C19 | 119 | 161 | 387 | 183 | 358 | 154 | 147 | 403 | 206 | 144 | 380 | 171 | 300 | 262 | 175 | 176 | 350 | 144 | 269 | 174 | 135 | 329 | 174 |
| Az Ext 01 32207-1504-H | 1-2 | C22 | 135 | 161 | 387 | 144 | 308 | 162 | 206 | 441 | 188 | 134 | 285 | 168 | 304 | 104 | 191 | 168 | 364 | 138 | 269 | 211 | 126 | 323 | 174 |
| Az Ext 02 32115-1012-A-B | 1-2 | C2 | 135 | 161 | 387 | 144 | 308 | 162 | 209 | 438 | 186 | 134 | 285 | 168 | 304 | 104 | 191 | 168 | 364 | 138 | 269 | 213 | 126 | 323 | 174 |
| Az Ext 02 32210-2701-A | 1-2 | C3 | 135 | 161 | 387 | 144 | 308 | 162 | 209 | 441 | 186 | 134 | 285 | 168 | 304 | 104 | 191 | 168 | 364 | 138 | 269 | 209 | 126 | 323 | 174 |
| Az Ext 02 32209-309-5 1/2-B-D | 1-2 | C4 | 135 | 161 | 387 | 144 | 308 | 165 | 206 | 455 | 186 | 134 | 285 | 168 | 304 | 104 | 191 | 168 | 364 | 141 | 269 | 209 | 126 | 323 | 174 |
| Az Ext 02 32208-3602-3609-J | 1-2 | C5 | 135 | 161 | 387 | 144 | 308 | 162 | 206 | 438 | 186 | 134 | 285 | 168 | 304 | 104 | 194 | 168 | 364 | 138 | 269 | 211 | 126 | 323 | 174 |
| Az Ext 02 31521-103-111-G | 1-2 | C6 | 135 | 161 | 387 | 144 | 308 | 162 | 206 | 403 | 186 | 134 | 285 | 168 | 304 | 104 | 191 | 168 | 364 | 138 | 269 | 211 | 126 | 323 | 174 |
| 2006 A/48 A | 1-1 | C27 | 119 | 161 | 387 | 183 | 361 | 154 | 147 | 421 | 209 | 144 | 380 | 171 | 300 | 262 | 175 | 176 | 350 | 147 | 269 | 176 | 135 | 329 | 174 |
| SSS'06 EP2-G | 1-1 | Son10 | 119 | 161 | 387 | 183 | 361 | 154 | 147 | 421 | 209 | 144 | 380 | 171 | 300 | 262 | 175 | 176 | 350 | 147 | 269 | 176 | 135 | 331 | 174 |
| SSS'06 A1-N | 1-1 | Son11 | 119 | 161 | 387 | 183 | 361 | 154 | 147 | 421 | 209 | 144 | 380 | 171 | 300 | 262 | 175 | 176 | 350 | 147 | 269 | 176 | 135 | 331 | 174 |
| SSS'06 Ya8-D | 1-1 | Son12 | 119 | 161 | 387 | 183 | 361 | 154 | 147 | 421 | 209 | 144 | 380 | 171 | 300 | 262 | 175 | 176 | 350 | 147 | 269 | 176 | 135 | 331 | 174 |
| SSS'06 A4-E | 1-1 | Son19 | 119 | 161 | 387 | 183 | 361 | 154 | 147 | 421 | 209 | 144 | 380 | 171 | 300 | 262 | 175 | 176 | 350 | 147 | 269 | 176 | 135 | 331 | 174 |
| SSS'06 T2-L | 1-1 | Son26 | 119 | 161 | 387 | 183 | 361 | 154 | 147 | 421 | 209 | 144 | 380 | 171 | 300 | 262 | 175 | 176 | 350 | 147 | 269 | 176 | 135 | 331 | 174 |
| SSS'06 C2-J | 1-1 | Son29 | 119 | 161 | 387 | 183 | 361 | 154 | 147 | 421 | 209 | 144 | 380 | 171 | 300 | 262 | 175 | 176 | 350 | 147 | 269 | 176 | 135 | 331 | 174 |
| SSS'06 Ye5-J | 1-1 | Son30 | 119 | 161 | 387 | 183 | 361 | 154 | 147 | 421 | 209 | 144 | 380 | 171 | 300 | 262 | 175 | 176 | 350 | 147 | 269 | 176 | 135 | 331 | 174 |
| SSS'06 EjES-M | 1-1 | Son32 | 119 | 161 | 387 | 183 | 361 | 154 | 147 | 421 | 209 | 144 | 380 | 171 | 300 | 262 | 175 | 176 | 350 | 147 | 269 | 176 | 135 | 331 | 174 |
| SSS'06 C3-M | 1-1 | Son36 | 119 | 161 | 387 | 183 | 361 | 154 | 147 | 421 | 209 | 144 | 380 | 171 | 300 | 262 | 175 | 176 | 350 | 147 | 269 | 176 | 135 | 331 | 174 |
| SSS'06 Ye2-A | 1-1 | Son37 | 119 | 161 | 387 | 183 | 361 | 154 | 147 | 421 | 209 | 144 | 380 | 171 | 300 | 262 | 175 | 176 | 350 | 147 | 269 | 176 | 135 | 331 | 174 |
| SSS'06 C1-A | 1-1 | Son38 | 119 | 161 | 387 | 183 | 361 | 154 | 147 | 421 | 209 | 144 | 380 | 171 | 300 | 262 | 175 | 176 | 350 | 147 | 269 | 176 | 135 | 331 | 174 |
| SSS'06 Nuri-C | 1-1 | Son39 | 119 | 161 | 387 | 183 | 361 | 154 | 147 | 421 | 209 | 144 | 380 | 171 | 300 | 262 | 175 | 176 | 350 | 147 | 269 | 176 | 135 | 331 | 174 |
| SSS'06 EP1-N | 1-1 | Son40 | 119 | 161 | 387 | 183 | 361 | 154 | 147 | 421 | 209 | 144 | 380 | 171 | 300 | 262 | 175 | 176 | 350 | 147 | 269 | 176 | 135 | 331 | 174 |
| SSS'06 T1-G | 1-1 | Son41 | 119 | 161 | 387 | 183 | 361 | 154 | 147 | 421 | 209 | 144 | 380 | 171 | 300 | 262 | 175 | 176 | 350 | 147 | 269 | 176 | 135 | 331 | 174 |
| SSS'06 Ya2-F | 1-1 | Son42 | 119 | 161 | 387 | 183 | 361 | 154 | 147 | 421 | 209 | 144 | 380 | 171 | 300 | 262 | 175 | 176 | 350 | 147 | 269 | 176 | 135 | 331 | 174 |
| SSS'06 Ya5-B | 1-1 | Son43 | 119 | 161 | 387 | 183 | 361 | 154 | 147 | 421 | 209 | 144 | 380 | 171 | 300 | 262 | 175 | 176 | 350 | 147 | 269 | 176 | 135 | 331 | 174 |
| SSS'06 Ye1-B | 1-1 | Son44 | 119 | 161 | 387 | 183 | 361 | 154 | 147 | 421 | 209 | 144 | 380 | 171 | 300 | 262 | 175 | 176 | 350 | 147 | 269 | 176 | 135 | 331 | 174 |
| SSS'06 Ya7-C | 1-1 | Son45 | 119 | 161 | 387 | 183 | 361 | 154 | 147 | 421 | 209 | 144 | 380 | 171 | 300 | 262 | 175 | 176 | 350 | 147 | 269 | 176 | 135 | 331 | 174 |
| SSS'06 Ya1-B | 1-1 | Son46 | 119 | 161 | 387 | 183 | 361 | 154 | 147 | 421 | 209 | 144 | 380 | 171 | 300 | 262 | 175 | 176 | 350 | 147 | 269 | 176 | 135 | 331 | 174 |
| SSS'06 A3-B | 1-1 | Son47 | 119 | 161 | 387 | 183 | 361 | 154 | 147 | 421 | 209 | 144 | 380 | 171 | 300 | 262 | 175 | 176 | 350 | 147 | 269 | 176 | 135 | 331 | 174 |
| SSS'06 Ya3-G | 1-1 | Son48 | 119 | 161 | 387 | 183 | 361 | 154 | 147 | 421 | 209 | 144 | 380 | 171 | 300 | 262 | 175 | 176 | 350 | 147 | 269 | 176 | 135 | 331 | 174 |
| SSS'06 Ya6-D | 1-1 | Son49 | 119 | 161 | 387 | 183 | 361 | 154 | 147 | 421 | 209 | 144 | 380 | 171 | 300 | 262 | 175 | 176 | 350 | 147 | 269 | 176 | 135 | 331 | 174 |
| SSS'06 Ye3-A | 1-1 | Son50 | 119 | 161 | 387 | 183 | 361 | 154 | 147 | 421 | 209 | 144 | 380 | 171 | 300 | 262 | 175 | 176 | 350 | 147 | 269 | 176 | 135 | 331 | 174 |
| SSS'06 NA-I | 1-1 | Son51 | 119 | 161 | 387 | 183 | 361 | 154 | 147 | 421 | 209 | 144 | 380 | 171 | 300 | 262 | 175 | 176 | 350 | 147 | 269 | 176 | 135 | 331 | 174 |
| SSS'06 Ye4-E | 1-1 | Son52 | 119 | 161 | 387 | 183 | 361 | 154 | 147 | 421 | 209 | 144 | 380 | 171 | 300 | 262 | 175 | 176 | 350 | 147 | 269 | 176 | 135 | 331 | 174 |
| SSS'06 SON003 T1 | 1-1 | Son53 | 119 | 161 | 387 | 183 | 361 | 154 | 147 | 421 | 209 | 144 | 380 | 171 | 300 | 262 | 175 | 176 | 350 | 147 | 269 | 176 | 135 | 331 | 174 |
| SSS'06 SON003 T2 | 1-1 | Son54 | 119 | 161 | 387 | 183 | 361 | 154 | 147 | 421 | 209 | 144 | 380 | 171 | 300 | 262 | 175 | 176 | 350 | 147 | 269 | 176 | 135 | 331 | 174 |
| SSS'06 A2-B | 1-1 | Son55 | 119 | 161 | 387 | 183 | 361 | 154 | 147 | 421 | 209 | 144 | 380 | 171 | 300 | 262 | 175 | 176 | 350 | 147 | 269 | 176 | 135 | 331 | 174 |
| SSS'06 A2-C | 1-1 | Son56 | 119 | 161 | 387 | 183 | 361 | 154 | 147 | 421 | 209 | 144 | 380 | 171 | 300 | 262 | 175 | 176 | 350 | 147 | 269 | 176 | 135 | 331 | 174 |
| SSS'06 A2-D | 1-1 | Son57 | 119 | 161 | 387 | 183 | 361 | 154 | 147 | 421 | 209 | 144 | 380 | 171 | 300 | 262 | 175 | 176 | 350 | 147 | 269 | 176 | 135 | 331 | 174 |
| SSS'06 A2-E | 1-1 | Son58 | 119 | 161 | 387 | 183 | 361 | 154 | 147 | 421 | 209 | 144 | 380 | 171 | 300 | 262 | 175 | 176 | 350 | 147 | 269 | 176 | 135 | 331 | 174 |
| SSS'06 A2-H | 1-1 | Son59 | 119 | 161 | 387 | 183 | 361 | 154 | 147 | 421 | 209 | 144 | 380 | 171 | 300 | 262 | 175 | 176 | 350 | 147 | 269 | 176 | 135 | 331 | 174 |
| SSS'06 A2-I | 1-1 | Son60 | 119 | 161 | 387 | 183 | 361 | 154 | 147 | 421 | 209 | 144 | 380 | 171 | 300 | 262 | 175 | 176 | 350 | 147 | 269 | 176 | 135 | 331 | 174 |
| SSS'06 A2-J | 1-1 | Son61 | 119 | 161 | 387 | 183 | 361 | 154 | 147 | 421 | 209 | 144 | 380 | 171 | 300 | 262 | 175 | 176 | 350 | 147 | 269 | 176 | 135 | 331 | 174 |
| SSS'06 A2-K | 1-1 | Son62 | 119 | 161 | 387 | 183 | 361 | 154 | 147 | 421 | 209 | 144 | 380 | 171 | 300 | 262 | 175 | 176 | 350 | 147 | 269 | 176 | 135 | 331 | 174 |
| SSS'06 A2-L | 1-1 | Son63 | 119 | 161 | 387 | 183 | 361 | 154 | 147 | 421 | 209 | 144 | 380 | 171 | 300 | 262 | 175 | 176 | 350 | 147 | 269 | 176 | 135 | 331 | 174 |
| SSS'06 A2-M | 1-1 | Son64 | 119 | 161 | 387 | 183 | 361 | 154 | 147 | 421 | 209 | 144 | 380 | 171 | 300 | 262 | 175 | 176 | 350 | 147 | 269 | 176 | 135 | 331 | 174 |
| SSS'06 A2-N | 1-1 | Son65 | 119 | 161 | 387 | 183 | 361 | 154 | 147 | 421 | 209 | 144 | 380 | 171 | 300 | 262 | 175 | 176 | 350 | 147 | 269 | 176 | 135 | 331 | 174 |
| SSS'06 MN-E | 1-1 | Son8 | 119 | 161 | 387 | 183 | 361 | 154 | 147 | 421 | 209 | 144 | 380 | 171 | 300 | 262 | 175 | 176 | 350 | 147 | 269 | 176 | 135 | 331 | 174 |
| SSS'07 VY3-C | 1-1 | Son66 | 119 | 161 | 387 | 183 | 361 | 154 | 147 | 421 | 209 | 144 | 380 | 171 | 300 | 262 | 175 | 176 | 350 | 147 | 269 | 176 | 135 | 331 | 174 |
| AZSO06 K19-F | 1-2 | N1 | 135 | 161 | 387 | 144 | 308 | 162 | 213 | 452 | 188 | 132 | 285 | 168 | 304 | 104 | 191 | 168 | 364 | 138 | 269 | 223 | 126 | 323 | 174 |
| AZSO06 K26-E | 1-2 | N2 | 135 | 161 | 387 | 144 | 308 | 162 | 209 | 458 | 186 | 132 | 285 | 168 | 304 | 104 | 191 | 168 | 364 | 138 | 269 | 215 | 126 | 323 | 174 |
| AZSO06 J13-C | 1-2 | N3 | 135 | 161 | 387 | 144 | 308 | 162 | 206 | 441 | 186 | 132 | 285 | 168 | 304 | 104 | 191 | 168 | 364 | 138 | 269 | 213 | 126 | 323 | 174 |
| AZSO06 B50-O | 1-2 | N4 | 135 | 161 | 387 | 144 | 308 | 162 | 206 | 463 | 186 | 132 | 285 | 168 | 304 | 104 | 191 | 168 | 364 | 138 | 269 | 193 | 126 | 323 | 174 |
| AZSO06 I12-E | 1-2 | N5 | 135 | 164 | 387 | 144 | 308 | 162 | 206 | 450 | 186 | 132 | 288 | 168 | 304 | 104 | 191 | 168 | 364 | 138 | 269 | 213 | 126 | 323 | 174 |
| AZSO06 O3-M | 1-2 | N6 | 135 | 161 | 387 | 144 | 308 | 162 | 206 | 441 | 186 | 132 | 285 | 168 | 304 | 104 | 191 | 168 | 364 | 138 | 269 | 209 | 126 | 323 | 174 |
| AZSO06 K7-J | 1-2 | N7 | 135 | 161 | 387 | 144 | 308 | 162 | 213 | 441 | 186 | 132 | 285 | 168 | 304 | 104 | 191 | 168 | 364 | 138 | 269 | 209 | 126 | 323 | 174 |
| AZSO06 B53-D | 1-1 | N8 | 119 | 164 | 385 | 188 | 337 | 221 | 147 | 471 | 168 | 148 | 259 | 174 | 316 | 262 | 178 | 161 | 353 | 144 | 269 | 186 | 133 | 329 | 178 |
| AZSO06 A18-K | 1-2 | N10 | 135 | 161 | 387 | 144 | 308 | 162 | 206 | 444 | 186 | 132 | 285 | 168 | 304 | 104 | 191 | 168 | 364 | 138 | 269 | 209 | 126 | 323 | 174 |
| AZSO06 G7-O | 1-2 | N11 | 135 | 161 | 390 | 144 | 308 | 162 | 206 | 441 | 186 | 132 | 288 | 168 | 304 | 104 | 191 | 168 | 364 | 138 | 269 | 197 | 126 | 323 | 174 |
| NCB-S 19-E | 1-1 | C24 | 119 | 161 | 387 | 183 | 358 | 154 | 147 | 403 | 200 | 144 | 425 | 171 | 300 | 262 | 175 | 176 | 350 | 144 | 269 | 174 | 135 | 329 | 174 |
| SSS'07 NA2-O | 1-1 | C31 | 119 | 161 | 387 | 183 | 358 | 154 | 147 | 403 | 206 | 148 | 377 | 171 | 300 | 262 | 175 | 176 | 350 | 144 | 269 | 176 | 135 | 329 | 174 |
| SSS'07 EP2-A | 1-1 | Son67 | 119 | 141 | 399 | 188 | 373 | 218 | 151 | 218 | 168 | 132 | 444 | 177 | 310 | 262 | 175 | 176 | 353 | 144 | 269 | 165 | 133 | 325 | 174 |
| SSS'08 EP2-H | 1-1 | C26 | 119 | 161 | 387 | 183 | 358 | 154 | 147 | 403 | 206 | 144 | 372 | 171 | 300 | 262 | 175 | 176 | 350 | 144 | 269 | 176 | 135 | 329 | 174 |
| SSS'08 EjES2-H | 1-1 | C28 | 119 | 161 | 387 | 183 | 358 | 154 | 147 | 403 | 209 | 144 | 380 | 171 | 300 | 262 | 175 | 176 | 350 | 144 | 269 | 174 | 135 | 329 | 174 |
| SSS'08 EP2-L | 1-1 | C29 | 119 | 161 | 387 | 183 | 355 | 154 | 147 | 403 | 206 | 144 | 372 | 171 | 300 | 262 | 175 | 176 | 350 | 144 | 269 | 176 | 135 | 329 | 174 |
| SSS'08 EP2-A | 1-1 | C30 | 119 | 161 | 387 | 183 | 355 | 154 | 147 | 403 | 206 | 144 | 372 | 171 | 300 | 262 | 175 | 176 | 350 | 144 | 269 | 176 | 135 | 329 | 174 |

**Supplementary Table for Review 3.** Allele sizes of 20 SSR loci (Grubisha and Cotty, 2009) for members of *Aspergillus flavus* VCGs OD02, MR17, CG136, YV36, and YV150. YV150 includes clone-corrected haplotypes, 36 clones among the 82 examined isolates.

| **VCG** | **Isolate ID** | **AF8** | **AF10** | **AF11** | **AF13** | **AF16** | **AF17** | **AF22** | **AF25** | **AF27** | **AF28** | **AF31** | **AF33** | **AF34** | **AF42** | **AF43** | **AF53** | **AF54** | **AF55** | **AF63** | **AF66** |
| --- | --- | --- | --- | --- | --- | --- | --- | --- | --- | --- | --- | --- | --- | --- | --- | --- | --- | --- | --- | --- | --- |
| ODO2 | O_001T99 | 179 | 317 | 151 | 122 | 181 | 367 | 199 | 308 | 280 | 118 | 308 | 171 | 335 | 162 | 387 | 134 | 177 | 172 | 135 | 269 |
| ODO2 | O_002T99 | 173 | 311 | 147 | 157 | 181 | 367 | 199 | 308 | 280 | 118 | 308 | 171 | 332 | 162 | 387 | 134 | 177 | 172 | 135 | 269 |
| ODO2 | O_003T99 | 173 | 308 | 147 | 157 | 181 | 367 | 199 | 308 | 280 | 118 | 308 | 171 | 332 | 162 | 387 | 134 | 177 | 172 | 135 | 269 |
| ODO2 | O_004T99 | 173 | 317 | 147 | 122 | 181 | 367 | 199 | 308 | 280 | 118 | 308 | 171 | 335 | 165 | 387 | 134 | 177 | 172 | 135 | 269 |
| ODO2 | O_006T99 | 173 | 317 | 147 | 122 | 181 | 367 | 199 | 308 | 280 | 118 | 308 | 171 | 335 | 162 | 387 | 134 | 177 | 172 | 135 | 269 |
| ODO2 | O_007T99 | 173 | 311 | 147 | 157 | 181 | 367 | 199 | 308 | 283 | 115 | 308 | 171 | 335 | 165 | 387 | 134 | 177 | 172 | 135 | 271 |
| ODO2 | O_009T99 | 173 | 348 | 147 | 157 | 181 | 367 | 199 | 311 | 283 | 118 | 308 | 171 | 332 | 162 | 387 | 134 | 177 | 172 | 135 | 269 |
| ODO2 | O_011T99 | 173 | 314 | 147 | 157 | 181 | 367 | 199 | 308 | 280 | 118 | 308 | 171 | 332 | 162 | 390 | 134 | 177 | 172 | 135 | 269 |
| ODO2 | O_013T99 | 173 | 317 | 147 | 122 | 181 | 367 | 199 | 308 | 280 | 118 | 308 | 171 | 339 | 162 | 387 | 134 | 177 | 172 | 135 | 269 |
| ODO2 | O_014T99 | 173 | 320 | 147 | 122 | 181 | 367 | 199 | 308 | 280 | 118 | 308 | 171 | 335 | 162 | 387 | 134 | 177 | 172 | 135 | 269 |
| ODO2 | O_015T99 | 173 | 311 | 147 | 157 | 181 | 367 | 199 | 308 | 283 | 118 | 308 | 171 | 332 | 162 | 387 | 134 | 177 | 172 | 135 | 269 |
| ODO2 | O_016T99 | 173 | 314 | 147 | 157 | 181 | 367 | 199 | 308 | 280 | 118 | 308 | 171 | 332 | 162 | 387 | 134 | 177 | 172 | 135 | 269 |
| ODO2 | O_041T00 | 176 | 308 | 147 | 157 | 181 | 367 | 199 | 308 | 280 | 118 | 308 | 171 | 335 | 162 | 387 | 134 | 177 | 172 | 135 | 269 |
| ODO2 | O_042T00 | 173 | 311 | 147 | 157 | 181 | 367 | 199 | 308 | 280 | 118 | 308 | 171 | 332 | 177 | 387 | 134 | 177 | 172 | 135 | 269 |
| ODO2 | O_043T00 | 173 | 314 | 147 | 157 | 181 | 373 | 199 | 308 | 280 | 118 | 308 | 171 | 332 | 165 | 390 | 134 | 177 | 172 | 135 | 269 |
| ODO2 | O_044T00 | 173 | 317 | 147 | 122 | 181 | 367 | 199 | 308 | 280 | 118 | 308 | 171 | 335 | 162 | 387 | 134 | 177 | 172 | 135 | 269 |
| ODO2 | O_045T00 | 173 | 290 | 147 | 157 | 181 | 367 | 199 | 308 | 280 | 118 | 308 | 171 | 332 | 162 | 390 | 134 | 177 | 172 | 135 | 269 |
| ODO2 | O_046T00 | 173 | 317 | 147 | 122 | 181 | 367 | 199 | 308 | 280 | 118 | 308 | 171 | 335 | 162 | 390 | 134 | 177 | 172 | 135 | 269 |
| ODO2 | O_047T00 | 173 | 314 | 147 | 160 | 181 | 370 | 199 | 308 | 283 | 118 | 308 | 171 | 335 | 165 | 387 | 134 | 181 | 172 | 135 | 271 |
| ODO2 | O_048T00 | 173 | 311 | 147 | 157 | 181 | 367 | 199 | 311 | 280 | 118 | 308 | 174 | 335 | 165 | 387 | 134 | 177 | 172 | 135 | 271 |
| ODO2 | O_049T00 | 173 | 317 | 147 | 122 | 181 | 367 | 199 | 308 | 280 | 118 | 308 | 171 | 335 | 162 | 387 | 134 | 177 | 172 | 135 | 269 |
| ODO2 | O_071T01 | 173 | 317 | 147 | 157 | 181 | 367 | 199 | 308 | 280 | 118 | 308 | 171 | 335 | 168 | 387 | 134 | 177 | 172 | 135 | 271 |
| ODO2 | O_072T01 | 173 | 317 | 147 | 122 | 181 | 367 | 199 | 308 | 280 | 118 | 308 | 171 | 335 | 162 | 387 | 134 | 177 | 172 | 135 | 269 |
| ODO2 | O_074T01 | 173 | 317 | 147 | 122 | 181 | 367 | 199 | 308 | 280 | 118 | 308 | 171 | 339 | 162 | 387 | 134 | 177 | 172 | 135 | 269 |
| ODO2 | O_076T01 | 173 | 317 | 147 | 122 | 181 | 367 | 199 | 308 | 280 | 118 | 308 | 171 | 332 | 162 | 387 | 134 | 177 | 172 | 135 | 269 |
| ODO2 | O_077T01 | 173 | 311 | 147 | 157 | 181 | 367 | 199 | 308 | 283 | 118 | 308 | 171 | 332 | 162 | 387 | 134 | 177 | 172 | 135 | 269 |
| ODO2 | O_078T01 | 173 | 317 | 147 | 157 | 181 | 367 | 199 | 308 | 280 | 118 | 308 | 171 | 332 | 162 | 387 | 134 | 177 | 172 | 135 | 269 |
| ODO2 | O_079T01 | 173 | 317 | 147 | 122 | 181 | 367 | 199 | 308 | 280 | 118 | 308 | 171 | 335 | 162 | 387 | 134 | 177 | 172 | 135 | 269 |
| ODO2 | O_080T01 | 173 | 311 | 147 | 160 | 181 | 367 | 199 | 308 | 280 | 118 | 308 | 171 | 335 | 165 | 387 | 134 | 177 | 172 | 135 | 271 |
| ODO2 | O_081T01 | 173 | 308 | 147 | 157 | 181 | 367 | 199 | 308 | 280 | 118 | 308 | 171 | 332 | 162 | 387 | 134 | 177 | 172 | 135 | 269 |
| ODO2 | O_082T01 | 173 | 290 | 147 | 157 | 181 | 367 | 199 | 308 | 280 | 118 | 308 | 171 | 332 | 162 | 390 | 134 | 177 | 172 | 135 | 269 |
| ODO2 | O_083T01 | 179 | 317 | 151 | 122 | 181 | 367 | 199 | 308 | 280 | 118 | 308 | 171 | 335 | 162 | 387 | 134 | 177 | 172 | 135 | 269 |
| ODO2 | O_105A00 | 173 | 279 | 147 | 160 | 181 | 367 | 199 | 308 | 280 | 118 | 308 | 171 | 332 | 168 | 390 | 134 | 177 | 172 | 135 | 269 |
| ODO2 | O_106A00 | 173 | 311 | 123 | 157 | 181 | 367 | 199 | 308 | 280 | 118 | 308 | 171 | 335 | 165 | 387 | 134 | 177 | 172 | 135 | 271 |
| ODO2 | O_107A00 | 173 | 314 | 147 | 157 | 181 | 367 | 199 | 308 | 283 | 118 | 308 | 171 | 332 | 162 | 387 | 134 | 177 | 172 | 135 | 269 |
| ODO2 | O_108A00 | 173 | 290 | 147 | 157 | 181 | 367 | 199 | 308 | 280 | 118 | 308 | 171 | 332 | 162 | 390 | 134 | 177 | 172 | 135 | 269 |
| ODO2 | O_110A00 | 173 | 308 | 147 | 157 | 184 | 367 | 199 | 308 | 280 | 118 | 308 | 171 | 332 | 162 | 387 | 134 | 177 | 172 | 135 | 269 |
| ODO2 | O_112A00 | 173 | 290 | 147 | 157 | 184 | 367 | 199 | 308 | 280 | 118 | 308 | 171 | 332 | 162 | 390 | 134 | 177 | 172 | 135 | 269 |
| ODO2 | O_113A00 | 173 | 279 | 147 | 160 | 181 | 367 | 199 | 311 | 280 | 118 | 308 | 171 | 332 | 171 | 387 | 134 | 177 | 172 | 135 | 269 |
| ODO2 | O_114A00 | 173 | 279 | 147 | 157 | 181 | 367 | 199 | 308 | 280 | 118 | 308 | 171 | 332 | 168 | 387 | 134 | 177 | 172 | 135 | 269 |
| ODO2 | O_115A00 | 173 | 279 | 147 | 157 | 184 | 367 | 199 | 308 | 280 | 118 | 308 | 171 | 332 | 171 | 387 | 134 | 177 | 172 | 135 | 269 |
| ODO2 | O_155A01 | 173 | 308 | 147 | 157 | 181 | 367 | 199 | 308 | 280 | 118 | 308 | 171 | 332 | 162 | 384 | 134 | 177 | 172 | 135 | 269 |
| ODO2 | O_156A01 | 173 | 305 | 147 | 160 | 181 | 367 | 199 | 308 | 280 | 118 | 308 | 171 | 332 | 162 | 390 | 134 | 177 | 172 | 135 | 269 |
| ODO2 | O_157A01 | 173 | 311 | 147 | 157 | 181 | 367 | 199 | 308 | 283 | 118 | 308 | 171 | 332 | 162 | 387 | 134 | 177 | 172 | 135 | 269 |
| ODO2 | O_158A01 | 173 | 290 | 147 | 157 | 181 | 367 | 199 | 308 | 280 | 118 | 308 | 171 | 332 | 162 | 390 | 134 | 177 | 172 | 135 | 269 |
| ODO2 | O_159A01 | 173 | 330 | 147 | 157 | 181 | 367 | 199 | 308 | 283 | 118 | 308 | 171 | 332 | 162 | 393 | 134 | 177 | 172 | 135 | 269 |
| ODO2 | O_160A01 | 173 | 311 | 147 | 157 | 181 | 367 | 199 | 308 | 280 | 118 | 308 | 171 | 342 | 162 | 387 | 134 | 177 | 172 | 135 | 269 |
| ODO2 | O_161A01 | 173 | 311 | 147 | 157 | 181 | 367 | 199 | 308 | 280 | 118 | 308 | 171 | 332 | 171 | 387 | 134 | 177 | 172 | 135 | 269 |
| ODO2 | O_162A01 | 173 | 314 | 147 | 160 | 181 | 367 | 199 | 308 | 280 | 118 | 308 | 171 | 332 | 165 | 390 | 134 | 177 | 172 | 135 | 269 |
| ODO2 | O_163A01 | 173 | 290 | 159 | 157 | 181 | 367 | 199 | 308 | 280 | 118 | 308 | 171 | 332 | 162 | 390 | 134 | 177 | 172 | 135 | 269 |
| ODO2 | O_164A01 | 173 | 290 | 147 | 157 | 181 | 367 | 199 | 308 | 280 | 118 | 308 | 171 | 329 | 162 | 390 | 134 | 177 | 172 | 135 | 269 |
| ODO2 | O_210A02 | 173 | 279 | 147 | 157 | 181 | 367 | 199 | 308 | 280 | 118 | 308 | 171 | 332 | 168 | 387 | 134 | 177 | 172 | 135 | 269 |
| ODO2 | O_211A02 | 173 | 314 | 147 | 157 | 181 | 367 | 199 | 308 | 280 | 118 | 308 | 174 | 332 | 162 | 387 | 134 | 177 | 172 | 135 | 269 |
| ODO2 | O_212A02 | 173 | 311 | 147 | 157 | 181 | 367 | 199 | 308 | 283 | 118 | 308 | 171 | 332 | 162 | 387 | 134 | 177 | 172 | 135 | 269 |
| ODO2 | O_213A02 | 173 | 311 | 147 | 157 | 181 | 367 | 199 | 308 | 280 | 118 | 308 | 171 | 332 | 162 | 387 | 134 | 177 | 172 | 135 | 269 |
| ODO2 | O_214A02 | 173 | 311 | 147 | 157 | 181 | 367 | 199 | 308 | 280 | 118 | 308 | 171 | 332 | 162 | 387 | 134 | 177 | 172 | 135 | 269 |
| ODO2 | O_215A02 | 173 | 308 | 147 | 157 | 181 | 367 | 199 | 308 | 280 | 118 | 308 | 171 | 332 | 162 | 387 | 134 | 177 | 172 | 135 | 269 |
| ODO2 | O_216A02 | 173 | 311 | 147 | 157 | 181 | 367 | 199 | 308 | 280 | 118 | 308 | 171 | 332 | 162 | 387 | 134 | 177 | 172 | 135 | 269 |
| ODO2 | O_217A02 | 173 | 279 | 147 | 157 | 181 | 367 | 199 | 308 | 280 | 118 | 308 | 171 | 332 | 168 | 384 | 134 | 177 | 172 | 135 | 269 |
| ODO2 | O_218A02 | 176 | 279 | 147 | 157 | 181 | 367 | 199 | 308 | 280 | 118 | 311 | 171 | 332 | 168 | 387 | 134 | 177 | 172 | 135 | 269 |
| ODO2 | O_219A02 | 173 | 314 | 147 | 157 | 181 | 367 | 199 | 308 | 283 | 118 | 308 | 171 | 332 | 162 | 387 | 134 | 177 | 172 | 135 | 269 |
| MR17 | V_031T99 | 194 | 398 | 203 | 125 | 175 | 382 | 183 | 329 | 262 | 135 | 372 | 174 | 300 | 230 | 387 | 147 | 173 | 190 | 131 | 269 |
| MR17 | V_033T99 | 194 | 386 | 203 | 125 | 175 | 382 | 183 | 329 | 262 | 135 | 366 | 174 | 300 | 218 | 387 | 144 | 173 | 190 | 131 | 269 |
| MR17 | V_034T99 | 194 | 404 | 203 | 125 | 175 | 385 | 183 | 329 | 262 | 135 | 363 | 174 | 300 | 221 | 387 | 144 | 173 | 190 | 131 | 269 |
| MR17 | V_035T99 | 200 | 410 | 209 | 125 | 175 | 382 | 183 | 329 | 262 | 135 | 372 | 174 | 300 | 227 | 387 | 147 | 173 | 190 | 131 | 269 |
| MR17 | V_036T99 | 197 | 413 | 206 | 125 | 175 | 382 | 183 | 329 | 262 | 135 | 375 | 174 | 300 | 227 | 387 | 147 | 173 | 190 | 131 | 269 |
| MR17 | V_037T99 | 194 | 336 | 203 | 125 | 175 | 382 | 183 | 329 | 262 | 135 | 363 | 174 | 300 | 218 | 387 | 144 | 173 | 190 | 131 | 269 |
| MR17 | V_039T99 | 194 | 404 | 209 | 125 | 175 | 382 | 183 | 329 | 262 | 135 | 363 | 174 | 300 | 218 | 387 | 144 | 173 | 190 | 131 | 269 |
| MR17 | V_040T99 | 185 | 383 | 206 | 125 | 175 | 382 | 183 | 329 | 262 | 135 | 375 | 174 | 300 | 221 | 387 | 144 | 173 | 190 | 131 | 269 |
| MR17 | V_067T00 | 197 | 401 | 203 | 125 | 175 | 382 | 183 | 329 | 262 | 135 | 384 | 174 | 300 | 230 | 387 | 147 | 173 | 190 | 131 | 269 |
| MR17 | V_068T00 | 194 | 395 | 206 | 125 | 175 | 388 | 183 | 329 | 262 | 135 | 363 | 174 | 300 | 218 | 387 | 144 | 173 | 190 | 131 | 269 |
| MR17 | V_069T00 | 194 | 401 | 206 | 125 | 175 | 358 | 183 | 329 | 262 | 135 | 372 | 174 | 300 | 227 | 387 | 147 | 173 | 192 | 131 | 269 |
| MR17 | V_070T00 | 194 | 401 | 203 | 125 | 175 | 397 | 183 | 329 | 262 | 135 | 372 | 174 | 300 | 227 | 384 | 147 | 173 | 190 | 131 | 269 |
| MR17 | V_097T01 | 194 | 401 | 203 | 125 | 175 | 382 | 183 | 329 | 262 | 135 | 363 | 174 | 300 | 218 | 387 | 144 | 173 | 190 | 131 | 269 |
| MR17 | V_098T01 | 194 | 413 | 206 | 125 | 175 | 382 | 183 | 329 | 262 | 135 | 363 | 174 | 300 | 221 | 387 | 144 | 173 | 190 | 131 | 269 |
| MR17 | V_099T01 | 203 | 317 | 203 | 125 | 175 | 382 | 183 | 329 | 262 | 135 | 366 | 174 | 300 | 159 | 387 | 144 | 173 | 192 | 131 | 269 |
| MR17 | V_100T01 | 194 | 348 | 203 | 125 | 175 | 382 | 183 | 329 | 262 | 135 | 366 | 174 | 300 | 221 | 387 | 144 | 173 | 192 | 131 | 269 |
| MR17 | V_101T01 | 194 | 407 | 203 | 125 | 175 | 385 | 183 | 329 | 262 | 135 | 372 | 174 | 300 | 227 | 387 | 147 | 173 | 190 | 131 | 269 |
| MR17 | V_102T01 | 194 | 380 | 206 | 125 | 175 | 382 | 183 | 329 | 262 | 135 | 369 | 174 | 300 | 221 | 387 | 144 | 173 | 190 | 131 | 269 |
| MR17 | V_104T01 | 197 | 401 | 203 | 125 | 175 | 382 | 183 | 329 | 262 | 135 | 327 | 174 | 300 | 218 | 387 | 144 | 173 | 190 | 131 | 269 |
| MR17 | V_138A00 | 212 | 407 | 203 | 125 | 175 | 382 | 183 | 329 | 262 | 135 | 363 | 174 | 300 | 218 | 387 | 144 | 173 | 190 | 131 | 269 |
| MR17 | V_139A00 | 194 | 380 | 206 | 125 | 175 | 382 | 183 | 329 | 262 | 135 | 369 | 174 | 300 | 218 | 387 | 144 | 177 | 190 | 131 | 269 |
| MR17 | V_140A00 | 200 | 404 | 203 | 125 | 175 | 382 | 183 | 329 | 262 | 135 | 414 | 174 | 300 | 236 | 387 | 147 | 173 | 190 | 131 | 269 |
| MR17 | V_141A00 | 194 | 395 | 200 | 125 | 175 | 382 | 183 | 329 | 262 | 135 | 363 | 174 | 300 | 218 | 387 | 144 | 173 | 190 | 131 | 269 |
| MR17 | V_142A00 | 194 | 398 | 203 | 125 | 175 | 382 | 183 | 329 | 262 | 135 | 372 | 174 | 300 | 227 | 387 | 147 | 173 | 190 | 131 | 269 |
| MR17 | V_143A00 | 194 | 401 | 203 | 125 | 175 | 385 | 183 | 329 | 262 | 135 | 366 | 174 | 300 | 208 | 387 | 144 | 173 | 190 | 131 | 269 |
| MR17 | V_144A00 | 157 | 293 | 209 | 125 | 175 | 382 | 183 | 329 | 262 | 135 | 363 | 174 | 300 | 218 | 387 | 144 | 173 | 190 | 131 | 269 |
| MR17 | V_145A00 | 200 | 401 | 209 | 125 | 175 | 382 | 183 | 329 | 262 | 135 | 372 | 174 | 300 | 230 | 387 | 147 | 173 | 190 | 131 | 269 |
| MR17 | V_146A00 | 212 | 398 | 203 | 125 | 175 | 382 | 183 | 329 | 262 | 135 | 363 | 174 | 300 | 218 | 387 | 144 | 173 | 190 | 131 | 269 |
| MR17 | V_147A00 | 194 | 380 | 206 | 125 | 175 | 382 | 183 | 329 | 262 | 135 | 369 | 174 | 300 | 218 | 390 | 144 | 173 | 190 | 131 | 269 |
| MR17 | V_148A00 | 194 | 395 | 209 | 125 | 175 | 382 | 183 | 329 | 262 | 135 | 366 | 174 | 300 | 192 | 387 | 144 | 173 | 190 | 131 | 269 |
| MR17 | V_150A00 | 194 | 389 | 203 | 125 | 175 | 382 | 183 | 329 | 262 | 135 | 369 | 174 | 300 | 218 | 387 | 144 | 173 | 190 | 131 | 269 |
| MR17 | V_151A00 | 194 | 404 | 206 | 125 | 175 | 382 | 183 | 329 | 262 | 135 | 363 | 174 | 300 | 218 | 387 | 144 | 173 | 190 | 131 | 269 |
| MR17 | V_152A00 | 197 | 401 | 203 | 125 | 175 | 382 | 183 | 329 | 262 | 135 | 372 | 174 | 300 | 227 | 387 | 147 | 173 | 190 | 131 | 269 |
| MR17 | V_153A00 | 212 | 395 | 209 | 125 | 175 | 385 | 183 | 329 | 262 | 135 | 363 | 174 | 300 | 224 | 387 | 144 | 173 | 190 | 131 | 269 |
| MR17 | V_154A00 | 194 | 383 | 206 | 125 | 175 | 382 | 183 | 329 | 262 | 135 | 369 | 174 | 300 | 218 | 387 | 144 | 173 | 190 | 131 | 269 |
| MR17 | V_185A01 | 200 | 410 | 203 | 125 | 175 | 382 | 191 | 329 | 262 | 135 | 375 | 174 | 300 | 227 | 387 | 147 | 173 | 190 | 131 | 269 |
| MR17 | V_186A01 | 194 | 407 | 206 | 125 | 175 | 382 | 183 | 329 | 262 | 135 | 363 | 174 | 300 | 221 | 387 | 144 | 173 | 190 | 131 | 269 |
| MR17 | V_187A01 | 200 | 404 | 209 | 125 | 175 | 382 | 183 | 329 | 262 | 135 | 372 | 174 | 300 | 227 | 387 | 147 | 173 | 190 | 131 | 269 |
| MR17 | V_189A01 | 194 | 392 | 206 | 125 | 175 | 382 | 183 | 329 | 262 | 135 | 369 | 174 | 300 | 218 | 387 | 144 | 173 | 190 | 131 | 269 |
| MR17 | V_191A01 | 197 | 351 | 203 | 125 | 175 | 382 | 183 | 329 | 262 | 135 | 360 | 174 | 300 | 221 | 387 | 144 | 173 | 190 | 131 | 269 |
| MR17 | V_192A01 | 194 | 401 | 212 | 125 | 175 | 382 | 183 | 329 | 262 | 135 | 372 | 174 | 300 | 230 | 387 | 147 | 173 | 192 | 131 | 269 |
| MR17 | V_193A01 | 194 | 398 | 200 | 125 | 175 | 382 | 183 | 329 | 262 | 135 | 372 | 174 | 300 | 218 | 387 | 144 | 173 | 190 | 131 | 269 |
| MR17 | V_194A01 | 197 | 401 | 203 | 125 | 175 | 382 | 183 | 329 | 262 | 135 | 390 | 174 | 300 | 227 | 387 | 147 | 173 | 190 | 131 | 269 |
| MR17 | V_196A01 | 194 | 395 | 203 | 125 | 175 | 382 | 183 | 329 | 262 | 135 | 363 | 174 | 300 | 218 | 387 | 144 | 173 | 190 | 131 | 269 |
| MR17 | V_197A01 | 194 | 398 | 203 | 125 | 175 | 382 | 183 | 329 | 262 | 135 | 363 | 174 | 300 | 218 | 387 | 144 | 173 | 190 | 131 | 269 |
| MR17 | V_198A01 | 194 | 401 | 200 | 125 | 175 | 382 | 183 | 329 | 262 | 138 | 363 | 174 | 300 | 218 | 387 | 144 | 173 | 190 | 131 | 269 |
| MR17 | V_199A01 | 194 | 401 | 224 | 125 | 175 | 382 | 183 | 329 | 262 | 135 | 372 | 174 | 300 | 230 | 387 | 147 | 173 | 190 | 131 | 269 |
| MR17 | V_200A01 | 194 | 404 | 212 | 125 | 175 | 382 | 183 | 329 | 262 | 135 | 375 | 174 | 300 | 239 | 387 | 147 | 173 | 190 | 131 | 269 |
| MR17 | V_201A01 | 194 | 398 | 206 | 125 | 175 | 382 | 183 | 329 | 262 | 135 | 363 | 174 | 300 | 218 | 387 | 144 | 173 | 190 | 131 | 269 |
| MR17 | V_202A01 | 197 | 401 | 203 | 125 | 175 | 382 | 183 | 329 | 262 | 135 | 378 | 174 | 300 | 227 | 387 | 147 | 173 | 190 | 131 | 269 |
| MR17 | V_203A01 | 200 | 398 | 206 | 125 | 175 | 382 | 183 | 329 | 262 | 135 | 372 | 174 | 300 | 236 | 387 | 147 | 173 | 190 | 131 | 269 |
| MR17 | V_204A01 | 194 | 404 | 203 | 125 | 175 | 382 | 183 | 329 | 262 | 135 | 372 | 174 | 300 | 227 | 387 | 147 | 173 | 190 | 131 | 269 |
| MR17 | V_205A01 | 200 | 401 | 203 | 125 | 175 | 382 | 183 | 329 | 262 | 135 | 372 | 174 | 300 | 233 | 387 | 147 | 173 | 190 | 131 | 269 |
| MR17 | V_206A01 | 203 | 407 | 230 | 125 | 175 | 382 | 183 | 329 | 262 | 135 | 369 | 174 | 300 | 218 | 387 | 144 | 173 | 192 | 131 | 269 |
| MR17 | V_207A01 | 197 | 383 | 203 | 125 | 175 | 382 | 183 | 329 | 262 | 135 | 369 | 174 | 300 | 218 | 387 | 144 | 173 | 190 | 131 | 269 |
| MR17 | V_208A01 | 194 | 398 | 206 | 125 | 175 | 382 | 183 | 329 | 262 | 135 | 375 | 174 | 300 | 227 | 387 | 147 | 173 | 190 | 131 | 269 |
| MR17 | V_209A01 | 197 | 398 | 203 | 125 | 175 | 382 | 183 | 329 | 262 | 135 | 372 | 174 | 300 | 227 | 387 | 147 | 173 | 190 | 131 | 269 |
| MR17 | V_232A02 | 194 | 401 | 203 | 125 | 175 | 382 | 183 | 329 | 262 | 135 | 375 | 174 | 300 | 227 | 387 | 147 | 173 | 190 | 131 | 269 |
| MR17 | V_233A02 | 194 | 395 | 203 | 125 | 175 | 382 | 183 | 329 | 262 | 135 | 372 | 174 | 300 | 218 | 387 | 144 | 173 | 190 | 131 | 269 |
| MR17 | V_234A02 | 197 | 354 | 206 | 125 | 175 | 385 | 183 | 329 | 262 | 135 | 363 | 174 | 300 | 221 | 387 | 144 | 173 | 190 | 131 | 269 |
| MR17 | V_235A02 | 194 | 407 | 212 | 125 | 175 | 382 | 183 | 329 | 262 | 135 | 372 | 174 | 300 | 221 | 387 | 144 | 173 | 192 | 131 | 269 |
| MR17 | V_236A02 | 194 | 404 | 203 | 125 | 175 | 382 | 183 | 329 | 262 | 135 | 372 | 174 | 300 | 227 | 384 | 147 | 173 | 190 | 131 | 269 |
| MR17 | V_237A02 | 197 | 395 | 206 | 125 | 175 | 382 | 183 | 329 | 262 | 135 | 366 | 174 | 300 | 218 | 387 | 144 | 173 | 190 | 131 | 269 |
| MR17 | V_238A02 | 194 | 398 | 203 | 125 | 175 | 382 | 183 | 329 | 262 | 135 | 363 | 174 | 300 | 208 | 387 | 144 | 173 | 190 | 131 | 269 |
| MR17 | V_239A02 | 194 | 380 | 203 | 125 | 175 | 382 | 183 | 329 | 262 | 135 | 369 | 174 | 300 | 224 | 387 | 144 | 173 | 190 | 131 | 269 |
| MR17 | V_240A02 | 200 | 398 | 159 | 125 | 175 | 382 | 183 | 329 | 262 | 135 | 372 | 174 | 300 | 230 | 387 | 147 | 173 | 190 | 131 | 269 |
| MR17 | V_243A02 | 197 | 398 | 206 | 125 | 175 | 382 | 183 | 329 | 262 | 135 | 372 | 174 | 300 | 239 | 387 | 147 | 173 | 192 | 131 | 269 |
| MR17 | V_245A02 | 209 | 395 | 206 | 125 | 175 | 385 | 183 | 329 | 262 | 135 | 363 | 174 | 300 | 218 | 387 | 144 | 173 | 190 | 131 | 269 |
| MR17 | V_246A02 | 200 | 401 | 221 | 125 | 175 | 382 | 183 | 329 | 262 | 135 | 372 | 174 | 300 | 227 | 387 | 147 | 173 | 190 | 131 | 269 |
| MR17 | V_247A02 | 194 | 383 | 203 | 125 | 175 | 382 | 183 | 329 | 262 | 135 | 369 | 174 | 300 | 218 | 387 | 144 | 173 | 190 | 131 | 269 |
| MR17 | V_248A02 | 194 | 398 | 206 | 125 | 175 | 382 | 183 | 329 | 262 | 135 | 378 | 174 | 300 | 227 | 387 | 147 | 173 | 190 | 131 | 269 |
| MR17 | V_250A02 | 194 | 395 | 203 | 125 | 175 | 382 | 183 | 329 | 262 | 138 | 363 | 174 | 300 | 218 | 387 | 144 | 173 | 190 | 131 | 269 |
| MR17 | V_251A02 | 194 | 401 | 206 | 125 | 175 | 382 | 183 | 329 | 262 | 135 | 357 | 174 | 300 | 218 | 387 | 144 | 173 | 190 | 131 | 269 |
| MR17 | V_252A02 | 194 | 404 | 206 | 125 | 175 | 382 | 183 | 329 | 262 | 135 | 372 | 174 | 300 | 227 | 387 | 147 | 173 | 190 | 131 | 269 |
| MR17 | V_253A02 | 194 | 398 | 203 | 125 | 175 | 382 | 183 | 329 | 262 | 135 | 375 | 174 | 300 | 230 | 387 | 147 | 173 | 190 | 131 | 269 |
| MR17 | V_254A02 | 203 | 401 | 177 | 125 | 175 | 382 | 183 | 329 | 262 | 135 | 372 | 174 | 300 | 227 | 387 | 147 | 173 | 190 | 131 | 269 |
| MR17 | V_255A02 | 188 | 357 | 212 | 125 | 175 | 382 | 183 | 329 | 262 | 135 | 363 | 174 | 300 | 218 | 387 | 144 | 173 | 190 | 131 | 269 |
| MR17 | V_256A02 | 194 | 380 | 209 | 125 | 175 | 382 | 183 | 329 | 262 | 135 | 333 | 174 | 300 | 218 | 387 | 144 | 173 | 190 | 131 | 269 |
| MR17 | V_257A02 | 206 | 380 | 203 | 125 | 175 | 382 | 183 | 329 | 262 | 135 | 369 | 174 | 300 | 218 | 387 | 144 | 173 | 190 | 131 | 269 |
| MR17 | V_258A02 | 194 | 354 | 203 | 125 | 175 | 385 | 183 | 329 | 262 | 135 | 369 | 174 | 300 | 221 | 387 | 144 | 173 | 190 | 131 | 269 |
| MR17 | V_259A02 | 197 | 401 | 156 | 125 | 175 | 382 | 183 | 329 | 262 | 135 | 366 | 174 | 300 | 218 | 387 | 144 | 173 | 190 | 131 | 269 |
| MR17 | V_260A02 | 197 | 383 | 209 | 125 | 175 | 382 | 183 | 329 | 262 | 135 | 369 | 174 | 300 | 218 | 387 | 144 | 173 | 190 | 131 | 269 |
| MR17 | V_261A02 | 194 | 404 | 171 | 125 | 175 | 382 | 183 | 329 | 262 | 135 | 396 | 174 | 300 | 183 | 387 | 144 | 173 | 190 | 131 | 269 |
| CG136 | U_018T99 | 176 | 282 | 206 | 160 | 190 | 353 | 179 | 305 | 262 | 141 | 324 | 174 | 316 | 189 | 387 | 150 | 161 | 182 | 127 | 269 |
| CG136 | U_019T99 | 182 | 282 | 200 | 160 | 187 | 353 | 179 | 305 | 262 | 141 | 321 | 174 | 319 | 189 | 384 | 150 | 161 | 180 | 127 | 269 |
| CG136 | U_021T99 | 182 | 282 | 200 | 160 | 187 | 353 | 179 | 305 | 262 | 141 | 321 | 174 | 316 | 189 | 384 | 150 | 161 | 180 | 127 | 269 |
| CG136 | U_022T99 | 176 | 285 | 212 | 145 | 190 | 353 | 179 | 305 | 262 | 141 | 321 | 174 | 316 | 189 | 384 | 150 | 161 | 180 | 127 | 269 |
| CG136 | U_023T99 | 176 | 282 | 206 | 160 | 190 | 353 | 179 | 305 | 262 | 141 | 321 | 174 | 316 | 192 | 384 | 150 | 161 | 180 | 127 | 269 |
| CG136 | U_024T99 | 176 | 282 | 132 | 160 | 190 | 353 | 179 | 305 | 262 | 141 | 321 | 174 | 316 | 189 | 384 | 150 | 161 | 180 | 127 | 269 |
| CG136 | U_025T99 | 176 | 282 | 215 | 160 | 190 | 353 | 179 | 305 | 262 | 121 | 321 | 174 | 316 | 189 | 384 | 150 | 161 | 182 | 127 | 269 |
| CG136 | U_027T99 | 176 | 305 | 206 | 160 | 190 | 356 | 179 | 305 | 262 | 141 | 321 | 174 | 316 | 189 | 384 | 150 | 161 | 180 | 127 | 269 |
| CG136 | U_028T99 | 176 | 282 | 206 | 163 | 190 | 353 | 179 | 305 | 262 | 141 | 321 | 174 | 316 | 192 | 384 | 150 | 161 | 180 | 127 | 269 |
| CG136 | U_029T99 | 176 | 282 | 215 | 160 | 190 | 353 | 179 | 305 | 262 | 138 | 321 | 174 | 316 | 189 | 384 | 172 | 161 | 180 | 127 | 269 |
| CG136 | U_030T99 | 176 | 282 | 215 | 160 | 190 | 353 | 179 | 305 | 262 | 141 | 321 | 174 | 316 | 189 | 384 | 150 | 161 | 180 | 127 | 269 |
| CG136 | U_052T00 | 176 | 282 | 218 | 160 | 190 | 353 | 179 | 305 | 262 | 141 | 321 | 174 | 316 | 189 | 384 | 150 | 161 | 180 | 127 | 269 |
| CG136 | U_054T00 | 179 | 282 | 206 | 160 | 190 | 353 | 179 | 305 | 262 | 141 | 321 | 174 | 316 | 189 | 384 | 150 | 161 | 180 | 127 | 269 |
| CG136 | U_055T00 | 176 | 282 | 185 | 157 | 190 | 353 | 179 | 305 | 262 | 141 | 321 | 174 | 316 | 189 | 384 | 150 | 161 | 180 | 127 | 269 |
| CG136 | U_057T00 | 176 | 282 | 188 | 160 | 190 | 353 | 179 | 305 | 262 | 141 | 321 | 174 | 316 | 162 | 384 | 150 | 161 | 180 | 127 | 269 |
| CG136 | U_059T00 | 176 | 282 | 209 | 160 | 190 | 353 | 179 | 305 | 262 | 141 | 321 | 174 | 319 | 189 | 384 | 150 | 161 | 180 | 127 | 269 |
| CG136 | U_061T00 | 176 | 282 | 159 | 160 | 190 | 353 | 179 | 305 | 262 | 141 | 321 | 174 | 316 | 189 | 384 | 150 | 161 | 180 | 127 | 269 |
| CG136 | U_062T00 | 176 | 282 | 188 | 160 | 190 | 353 | 179 | 305 | 262 | 141 | 321 | 174 | 316 | 192 | 384 | 150 | 161 | 180 | 127 | 269 |
| CG136 | U_065T00 | 160 | 287 | 212 | 160 | 193 | 353 | 179 | 305 | 262 | 141 | 321 | 174 | 316 | 189 | 384 | 150 | 161 | 180 | 127 | 269 |
| CG136 | U_066T00 | 176 | 282 | 212 | 160 | 190 | 353 | 179 | 305 | 262 | 141 | 321 | 174 | 316 | 208 | 384 | 150 | 161 | 180 | 127 | 269 |
| CG136 | U_084T01 | 176 | 282 | 200 | 163 | 190 | 353 | 179 | 305 | 262 | 141 | 321 | 174 | 316 | 214 | 384 | 150 | 161 | 180 | 127 | 269 |
| CG136 | U_086T01 | 176 | 282 | 209 | 160 | 190 | 353 | 179 | 305 | 262 | 141 | 321 | 174 | 316 | 189 | 384 | 150 | 161 | 180 | 127 | 269 |
| CG136 | U_087T01 | 179 | 285 | 209 | 160 | 190 | 353 | 179 | 305 | 262 | 141 | 321 | 174 | 316 | 189 | 384 | 150 | 161 | 180 | 127 | 269 |
| CG136 | U_089T01 | 176 | 293 | 215 | 160 | 190 | 353 | 179 | 305 | 262 | 141 | 321 | 174 | 316 | 189 | 384 | 150 | 161 | 180 | 127 | 269 |
| CG136 | U_092T01 | 176 | 285 | 200 | 160 | 190 | 353 | 179 | 305 | 262 | 141 | 321 | 177 | 316 | 189 | 384 | 150 | 161 | 180 | 127 | 269 |
| CG136 | U_094T01 | 176 | 282 | 206 | 160 | 190 | 353 | 179 | 305 | 262 | 138 | 321 | 174 | 316 | 189 | 384 | 150 | 161 | 182 | 127 | 269 |
| CG136 | U_095T01 | 176 | 282 | 227 | 160 | 190 | 353 | 179 | 305 | 262 | 141 | 321 | 174 | 316 | 189 | 384 | 150 | 161 | 182 | 127 | 269 |
| CG136 | U_096T01 | 176 | 282 | 182 | 151 | 193 | 353 | 179 | 305 | 262 | 141 | 321 | 174 | 316 | 189 | 384 | 150 | 161 | 180 | 127 | 269 |
| CG136 | U_117A00 | 179 | 282 | 185 | 160 | 190 | 353 | 179 | 305 | 262 | 141 | 321 | 174 | 316 | 195 | 384 | 150 | 161 | 182 | 127 | 269 |
| CG136 | U_118A00 | 176 | 302 | 206 | 160 | 190 | 353 | 179 | 305 | 262 | 141 | 321 | 174 | 316 | 189 | 384 | 150 | 161 | 180 | 127 | 269 |
| CG136 | U_119A00 | 176 | 285 | 185 | 160 | 190 | 353 | 179 | 305 | 262 | 141 | 321 | 174 | 316 | 189 | 384 | 150 | 161 | 180 | 127 | 269 |
| CG136 | U_120A00 | 176 | 285 | 167 | 160 | 190 | 353 | 179 | 305 | 262 | 141 | 321 | 174 | 316 | 192 | 384 | 150 | 161 | 180 | 127 | 269 |
| CG136 | U_121A00 | 176 | 302 | 206 | 160 | 190 | 353 | 179 | 305 | 262 | 144 | 321 | 174 | 316 | 189 | 384 | 150 | 161 | 180 | 127 | 269 |
| CG136 | U_122A00 | 176 | 299 | 177 | 160 | 190 | 353 | 179 | 305 | 262 | 141 | 321 | 174 | 316 | 189 | 384 | 150 | 161 | 180 | 127 | 269 |
| CG136 | U_123A00 | 176 | 282 | 212 | 160 | 190 | 353 | 179 | 305 | 262 | 141 | 321 | 174 | 316 | 189 | 384 | 150 | 161 | 180 | 127 | 269 |
| CG136 | U_124A00 | 176 | 282 | 206 | 163 | 190 | 353 | 179 | 305 | 262 | 144 | 321 | 174 | 316 | 189 | 384 | 150 | 161 | 180 | 127 | 269 |
| CG136 | U_125A00 | 176 | 299 | 206 | 166 | 190 | 353 | 179 | 305 | 262 | 141 | 321 | 174 | 316 | 189 | 384 | 153 | 161 | 180 | 127 | 269 |
| CG136 | U_126A00 | 176 | 282 | 206 | 160 | 193 | 353 | 179 | 305 | 262 | 141 | 321 | 174 | 316 | 189 | 384 | 153 | 161 | 180 | 127 | 269 |
| CG136 | U_127A00 | 176 | 285 | 212 | 160 | 193 | 353 | 179 | 305 | 262 | 141 | 321 | 174 | 316 | 189 | 384 | 150 | 161 | 180 | 127 | 269 |
| CG136 | U_128A00 | 176 | 282 | 206 | 160 | 190 | 353 | 179 | 305 | 262 | 141 | 321 | 174 | 316 | 189 | 384 | 150 | 161 | 180 | 127 | 269 |
| CG136 | U_130A00 | 176 | 296 | 174 | 160 | 190 | 353 | 179 | 305 | 262 | 141 | 321 | 174 | 316 | 189 | 384 | 150 | 161 | 180 | 127 | 269 |
| CG136 | U_131A00 | 179 | 285 | 215 | 160 | 190 | 353 | 179 | 305 | 262 | 141 | 321 | 174 | 316 | 189 | 384 | 150 | 161 | 180 | 127 | 269 |
| CG136 | U_132A00 | 176 | 282 | 182 | 160 | 190 | 353 | 179 | 305 | 262 | 141 | 321 | 174 | 316 | 192 | 384 | 150 | 161 | 180 | 127 | 269 |
| CG136 | U_134A00 | 176 | 305 | 206 | 163 | 190 | 353 | 179 | 305 | 262 | 141 | 321 | 174 | 316 | 189 | 384 | 150 | 161 | 180 | 127 | 269 |
| CG136 | U_135A00 | 176 | 282 | 188 | 160 | 190 | 353 | 179 | 305 | 262 | 141 | 321 | 174 | 316 | 189 | 384 | 150 | 161 | 180 | 127 | 269 |
| CG136 | U_136A00 | 179 | 299 | 206 | 160 | 190 | 353 | 179 | 308 | 262 | 141 | 321 | 174 | 316 | 189 | 384 | 150 | 161 | 180 | 127 | 269 |
| CG136 | U_165A01 | 176 | 282 | 185 | 160 | 190 | 353 | 179 | 305 | 262 | 141 | 321 | 174 | 316 | 189 | 384 | 150 | 161 | 180 | 127 | 269 |
| CG136 | U_166A01 | 176 | 282 | 185 | 160 | 190 | 353 | 179 | 305 | 262 | 141 | 321 | 174 | 316 | 192 | 384 | 150 | 161 | 180 | 127 | 269 |
| CG136 | U_167A01 | 176 | 296 | 209 | 160 | 193 | 353 | 179 | 305 | 262 | 141 | 324 | 174 | 316 | 189 | 384 | 150 | 161 | 180 | 127 | 269 |
| CG136 | U_168A01 | 176 | 302 | 206 | 163 | 190 | 353 | 179 | 305 | 262 | 141 | 321 | 174 | 316 | 189 | 384 | 150 | 161 | 180 | 127 | 269 |
| CG136 | U_170A01 | 176 | 282 | 209 | 160 | 193 | 353 | 179 | 305 | 262 | 141 | 321 | 174 | 316 | 189 | 384 | 150 | 161 | 180 | 127 | 269 |
| CG136 | U_175A01 | 176 | 299 | 206 | 160 | 190 | 353 | 179 | 305 | 262 | 141 | 321 | 174 | 316 | 189 | 384 | 150 | 161 | 180 | 127 | 269 |
| CG136 | U_176A01 | 176 | 296 | 206 | 137 | 190 | 353 | 179 | 305 | 262 | 141 | 321 | 174 | 316 | 189 | 387 | 150 | 161 | 180 | 127 | 269 |
| CG136 | U_178A01 | 176 | 282 | 212 | 160 | 190 | 353 | 179 | 305 | 262 | 141 | 321 | 174 | 316 | 189 | 384 | 150 | 161 | 180 | 127 | 269 |
| CG136 | U_180A01 | 176 | 296 | 209 | 160 | 190 | 353 | 179 | 305 | 262 | 141 | 321 | 174 | 316 | 189 | 384 | 150 | 165 | 180 | 127 | 269 |
| CG136 | U_181A01 | 176 | 282 | 188 | 160 | 190 | 353 | 179 | 305 | 262 | 141 | 321 | 174 | 316 | 189 | 384 | 150 | 161 | 180 | 127 | 269 |
| CG136 | U_182A01 | 176 | 299 | 209 | 160 | 190 | 353 | 179 | 305 | 262 | 141 | 321 | 174 | 316 | 189 | 384 | 150 | 161 | 180 | 127 | 269 |
| CG136 | U_183A01 | 176 | 285 | 185 | 160 | 190 | 353 | 179 | 305 | 262 | 141 | 321 | 174 | 316 | 189 | 384 | 150 | 161 | 180 | 127 | 269 |
| CG136 | U_184A01 | 188 | 282 | 185 | 160 | 190 | 353 | 179 | 305 | 262 | 141 | 321 | 174 | 316 | 189 | 384 | 150 | 161 | 180 | 127 | 269 |
| CG136 | U_220A02 | 176 | 282 | 185 | 160 | 190 | 353 | 179 | 305 | 262 | 141 | 321 | 174 | 316 | 189 | 384 | 150 | 161 | 180 | 127 | 269 |
| CG136 | U_221A02 | 176 | 299 | 206 | 160 | 190 | 353 | 179 | 305 | 262 | 141 | 321 | 174 | 316 | 189 | 384 | 150 | 161 | 180 | 127 | 269 |
| CG136 | U_222A02 | 176 | 299 | 209 | 160 | 190 | 353 | 179 | 305 | 262 | 141 | 321 | 174 | 316 | 189 | 387 | 150 | 161 | 180 | 127 | 269 |
| CG136 | U_223A02 | 176 | 296 | 206 | 160 | 190 | 353 | 179 | 305 | 262 | 141 | 321 | 174 | 316 | 189 | 384 | 150 | 161 | 180 | 127 | 269 |
| CG136 | U_224A02 | 176 | 285 | 233 | 160 | 190 | 353 | 179 | 308 | 262 | 141 | 321 | 174 | 316 | 189 | 384 | 150 | 161 | 180 | 127 | 269 |
| CG136 | U_225A02 | 176 | 282 | 206 | 160 | 190 | 353 | 179 | 305 | 262 | 141 | 321 | 174 | 316 | 189 | 384 | 150 | 161 | 180 | 127 | 269 |
| CG136 | U_226A02 | 176 | 296 | 212 | 160 | 190 | 353 | 179 | 305 | 262 | 141 | 321 | 174 | 316 | 189 | 384 | 150 | 161 | 180 | 127 | 269 |
| CG136 | U_227A02 | 176 | 299 | 206 | 160 | 190 | 353 | 179 | 305 | 262 | 141 | 321 | 174 | 316 | 189 | 384 | 150 | 161 | 180 | 127 | 269 |
| CG136 | U_228A02 | 176 | 296 | 209 | 160 | 190 | 353 | 179 | 305 | 262 | 141 | 321 | 174 | 316 | 189 | 384 | 150 | 161 | 180 | 127 | 269 |
| CG136 | U_229A02 | 176 | 282 | 212 | 160 | 190 | 353 | 179 | 308 | 262 | 141 | 321 | 174 | 316 | 189 | 384 | 150 | 161 | 180 | 127 | 269 |
| CG136 | U_230A02 | 176 | 282 | 185 | 160 | 190 | 353 | 179 | 305 | 262 | 138 | 321 | 174 | 316 | 192 | 384 | 150 | 161 | 180 | 127 | 269 |
| CG136 | U_231A02 | 176 | 282 | 185 | 160 | 190 | 353 | 179 | 305 | 262 | 141 | 321 | 174 | 316 | 189 | 384 | 150 | 161 | 180 | 127 | 269 |
| YV36 | A462_AL91 | 179 | 285 | 162 | 160 | 190 | 353 | 187 | 304 | 274 | 118 | 308 | 171 | 310 | 162 | 387 | 134 | 169 | 174 | 135 | 269 |
| YV36 | A454_MS91 | 182 | 288 | 162 | 163 | 190 | 353 | 187 | 304 | 274 | 118 | 308 | 171 | 310 | 162 | 384 | 134 | 169 | 174 | 135 | 269 |
| YV36 | A450_AR91 | 179 | 285 | 162 | 163 | 190 | 353 | 187 | 304 | 274 | 118 | 308 | 171 | 310 | 162 | 384 | 134 | 169 | 174 | 135 | 269 |
| YV36 | A474_AZ92 | 176 | 285 | 182 | 160 | 190 | 353 | 187 | 304 | 274 | 118 | 308 | 171 | 310 | 162 | 384 | 134 | 169 | 174 | 135 | 269 |
| YV36 | A486_AZ93 | 176 | 285 | 165 | 166 | 190 | 353 | 187 | 304 | 274 | 118 | 308 | 171 | 310 | 162 | 384 | 134 | 169 | 174 | 135 | 269 |
| YV36 | A496_AZ97 | 176 | 285 | 138 | 160 | 190 | 353 | 187 | 304 | 274 | 118 | 308 | 171 | 310 | 162 | 384 | 134 | 169 | 174 | 135 | 269 |
| YV36 | A499_AZ97 | 176 | 288 | 162 | 163 | 190 | 353 | 187 | 304 | 274 | 118 | 308 | 171 | 310 | 162 | 384 | 134 | 169 | 174 | 135 | 269 |
| YV36 | A500_AZ97 | 179 | 285 | 162 | 160 | 190 | 353 | 187 | 304 | 274 | 118 | 308 | 171 | 310 | 162 | 384 | 134 | 169 | 174 | 135 | 269 |
| YV36 | A502_AZ97 | 176 | 288 | 162 | 160 | 190 | 353 | 187 | 307 | 274 | 118 | 308 | 171 | 310 | 162 | 384 | 134 | 169 | 174 | 135 | 269 |
| YV36 | A508_AZ97 | 176 | 299 | 162 | 160 | 190 | 353 | 187 | 304 | 274 | 118 | 308 | 171 | 310 | 162 | 384 | 134 | 169 | 174 | 135 | 269 |
| YV36 | A510_AZ97 | 176 | 285 | 162 | 160 | 190 | 353 | 187 | 304 | 274 | 118 | 308 | 171 | 310 | 165 | 384 | 134 | 169 | 174 | 135 | 269 |
| YV36 | A467_TX91 | 176 | 285 | 162 | 172 | 190 | 353 | 187 | 304 | 274 | 118 | 308 | 171 | 310 | 162 | 384 | 134 | 169 | 174 | 135 | 269 |
| YV36 | A705_TX99 | 179 | 285 | 162 | 166 | 190 | 353 | 187 | 304 | 274 | 118 | 308 | 171 | 310 | 162 | 384 | 134 | 169 | 174 | 135 | 269 |
| YV36 | A707_TX99 | 176 | 285 | 162 | 160 | 193 | 353 | 187 | 304 | 274 | 118 | 308 | 171 | 310 | 162 | 384 | 134 | 169 | 174 | 135 | 269 |
| YV36 | A673_TX00 | 176 | 285 | 156 | 160 | 190 | 353 | 187 | 304 | 274 | 118 | 308 | 171 | 310 | 162 | 384 | 134 | 169 | 174 | 135 | 269 |
| YV36 | A674_TX00 | 176 | 285 | 162 | 160 | 190 | 353 | 187 | 304 | 274 | 118 | 308 | 174 | 310 | 162 | 384 | 134 | 169 | 174 | 135 | 269 |
| YV36 | A678_TX00 | 176 | 285 | 165 | 163 | 190 | 353 | 187 | 304 | 274 | 118 | 308 | 171 | 310 | 162 | 384 | 134 | 169 | 174 | 135 | 269 |
| YV36 | A679_TX00 | 176 | 288 | 162 | 145 | 190 | 353 | 187 | 304 | 274 | 118 | 308 | 183 | 310 | 162 | 384 | 134 | 169 | 174 | 135 | 269 |
| YV36 | A685_TX00 | 176 | 288 | 162 | 160 | 190 | 353 | 187 | 304 | 274 | 118 | 308 | 171 | 310 | 165 | 384 | 134 | 169 | 174 | 135 | 269 |
| YV36 | A688_TX00 | 179 | 285 | 165 | 160 | 190 | 353 | 187 | 304 | 274 | 118 | 308 | 171 | 310 | 162 | 384 | 134 | 169 | 174 | 135 | 269 |
| YV36 | A689_TX00 | 176 | 285 | 162 | 160 | 190 | 353 | 187 | 304 | 274 | 118 | 311 | 171 | 310 | 162 | 384 | 134 | 169 | 174 | 135 | 269 |
| YV36 | A690_TX01 | 176 | 288 | 162 | 160 | 190 | 353 | 187 | 304 | 274 | 118 | 308 | 171 | 310 | 162 | 384 | 134 | 169 | 174 | 135 | 269 |
| YV36 | A693_TX01 | 176 | 288 | 162 | 160 | 190 | 353 | 187 | 304 | 274 | 118 | 308 | 171 | 310 | 168 | 384 | 134 | 169 | 174 | 135 | 269 |
| YV36 | A694_TX01 | 176 | 282 | 162 | 160 | 190 | 353 | 187 | 304 | 274 | 118 | 308 | 171 | 310 | 165 | 384 | 134 | 169 | 174 | 135 | 269 |
| YV36 | A695_TX01 | 176 | 288 | 156 | 160 | 190 | 353 | 187 | 304 | 274 | 118 | 308 | 171 | 310 | 162 | 384 | 134 | 169 | 174 | 135 | 269 |
| YV36 | A423_AZ02 | 176 | 285 | 174 | 160 | 190 | 353 | 187 | 304 | 274 | 118 | 308 | 171 | 310 | 165 | 384 | 134 | 169 | 174 | 135 | 269 |
| YV36 | A425_AZ02 | 179 | 285 | 162 | 166 | 190 | 353 | 187 | 304 | 274 | 118 | 308 | 171 | 310 | 165 | 384 | 134 | 169 | 174 | 135 | 269 |
| YV36 | A428_AZ02 | 176 | 285 | 165 | 160 | 193 | 353 | 187 | 304 | 274 | 118 | 308 | 171 | 310 | 162 | 384 | 134 | 169 | 174 | 135 | 269 |
| YV36 | A431_AZ02 | 176 | 285 | 162 | 151 | 190 | 353 | 187 | 304 | 274 | 118 | 308 | 171 | 310 | 162 | 384 | 134 | 169 | 174 | 135 | 269 |
| YV36 | A444_MX05 | 176 | 285 | 162 | 160 | 190 | 353 | 187 | 304 | 274 | 121 | 308 | 171 | 310 | 162 | 384 | 134 | 169 | 174 | 135 | 269 |
| YV36 | A446_MX05 | 179 | 288 | 162 | 160 | 190 | 353 | 187 | 304 | 274 | 118 | 308 | 171 | 310 | 162 | 384 | 134 | 169 | 174 | 135 | 269 |
| YV36 | A524_TX04 | 176 | 285 | 165 | 160 | 190 | 353 | 187 | 304 | 274 | 118 | 308 | 171 | 310 | 162 | 384 | 134 | 169 | 174 | 135 | 269 |
| YV36 | A596_TX05 | 176 | 285 | 162 | 160 | 190 | 353 | 187 | 304 | 274 | 118 | 308 | 171 | 310 | 162 | 387 | 134 | 169 | 174 | 135 | 269 |
| YV36 | A606_TX05 | 176 | 285 | 168 | 160 | 190 | 353 | 187 | 304 | 274 | 118 | 308 | 171 | 310 | 162 | 384 | 134 | 169 | 174 | 135 | 269 |
| YV36 | A622_TX05 | 176 | 288 | 165 | 163 | 190 | 353 | 187 | 304 | 274 | 118 | 308 | 171 | 310 | 162 | 384 | 134 | 169 | 174 | 135 | 269 |
| YV36 | A648_TX05 | 182 | 285 | 162 | 160 | 190 | 353 | 187 | 304 | 274 | 118 | 308 | 171 | 310 | 162 | 384 | 134 | 169 | 174 | 135 | 269 |
| YV36 | A671_TX05 | 176 | 285 | 162 | 160 | 190 | 353 | 187 | 304 | 274 | 118 | 308 | 171 | 310 | 162 | 384 | 134 | 169 | 174 | 135 | 269 |
| YV36 | A672_TX05 | 176 | 285 | 162 | 163 | 190 | 353 | 187 | 304 | 274 | 118 | 308 | 171 | 310 | 162 | 384 | 134 | 169 | 174 | 135 | 269 |
| YV36 | AF36_398_01 | 176 | 285 | 162 | 160 | 190 | 353 | 187 | 304 | 274 | 118 | 308 | 171 | 310 | 162 | 384 | 134 | 169 | 174 | 135 | 269 |
| YV150 1-1 | C19 | 206 | 380 | 144 | 161 | 175 | 350 | 183 | 323 | 262 | 118 | 358 | 171 | 300 | 154 | 387 | 144 | 176 | 174 | 135 | 269 |
| YV150 1-1 | C21 | 176 | 288 | 137 | 182 | 175 | 350 | 183 | 323 | 274 | 125 | 358 | 171 | 300 | 154 | 387 | 144 | 165 | 174 | 130 | 269 |
| YV150 1-1 | LG284 | 176 | 0 | 137 | 182 | 175 | 350 | 183 | 323 | 0 | 125 | 358 | 171 | 300 | 154 | 387 | 144 | 165 | 174 | 135 | 269 |
| YV150 1-1 | C18 | 209 | 372 | 144 | 161 | 175 | 350 | 183 | 330 | 262 | 118 | 355 | 171 | 300 | 154 | 387 | 144 | 176 | 174 | 135 | 269 |
| YV150 1-1 | C11 | 206 | 383 | 144 | 161 | 175 | 350 | 183 | 330 | 262 | 118 | 361 | 171 | 300 | 154 | 387 | 144 | 180 | 174 | 135 | 269 |
| YV150 1-1 | C24 | 200 | 425 | 144 | 161 | 175 | 350 | 183 | 330 | 262 | 118 | 358 | 171 | 300 | 154 | 387 | 144 | 176 | 190 | 135 | 269 |
| YV150 1-1 | Son10 | 209 | 380 | 147 | 161 | 175 | 350 | 183 | 330 | 262 | 118 | 361 | 171 | 300 | 154 | 387 | 144 | 176 | 174 | 135 | 269 |
| YV150 1-1 | C31 | 206 | 377 | 144 | 161 | 175 | 350 | 183 | 326 | 262 | 118 | 358 | 171 | 300 | 154 | 387 | 147 | 176 | 182 | 135 | 269 |
| YV150 1-1 | N72 | 206 | 377 | 144 | 161 | 175 | 350 | 183 | 330 | 262 | 118 | 361 | 171 | 300 | 154 | 387 | 144 | 176 | 174 | 135 | 269 |
| YV150 1-1 | C26 | 206 | 372 | 144 | 161 | 175 | 350 | 183 | 330 | 262 | 118 | 358 | 171 | 300 | 154 | 387 | 144 | 176 | 174 | 135 | 269 |
| YV150 1-1 | C28 | 209 | 380 | 144 | 161 | 175 | 350 | 183 | 330 | 262 | 118 | 358 | 171 | 300 | 154 | 387 | 144 | 176 | 174 | 135 | 269 |
| YV150 1-1 | C29 | 206 | 372 | 144 | 161 | 175 | 350 | 183 | 330 | 262 | 118 | 355 | 171 | 300 | 154 | 387 | 144 | 176 | 174 | 135 | 269 |
| YV150 1-2 | C34 | 186 | 285 | 137 | 161 | 191 | 364 | 144 | 323 | 104 | 134 | 308 | 168 | 304 | 162 | 387 | 134 | 168 | 174 | 126 | 269 |
| YV150 1-2 | C13 | 186 | 285 | 137 | 161 | 191 | 364 | 144 | 323 | 104 | 134 | 308 | 168 | 304 | 162 | 390 | 134 | 168 | 174 | 126 | 269 |
| YV150 1-2 | C15 | 186 | 285 | 137 | 167 | 191 | 364 | 144 | 323 | 104 | 134 | 308 | 168 | 304 | 162 | 387 | 134 | 168 | 174 | 126 | 269 |
| YV150 1-2 | C12 | 188 | 285 | 137 | 161 | 191 | 364 | 144 | 323 | 104 | 134 | 308 | 168 | 304 | 162 | 387 | 134 | 168 | 174 | 126 | 269 |
| YV150 1-2 | C16 | 186 | 285 | 137 | 157 | 191 | 364 | 144 | 323 | 104 | 134 | 308 | 168 | 304 | 162 | 387 | 134 | 168 | 174 | 126 | 269 |
| YV150 1-2 | C20 | 186 | 285 | 137 | 161 | 191 | 364 | 144 | 323 | 104 | 134 | 308 | 168 | 304 | 162 | 387 | 134 | 168 | 174 | 126 | 269 |
| YV150 1-2 | C7 | 186 | 285 | 137 | 161 | 191 | 364 | 144 | 323 | 104 | 134 | 308 | 168 | 304 | 165 | 387 | 134 | 168 | 174 | 126 | 269 |
| YV150 1-2 | C8 | 186 | 285 | 137 | 161 | 191 | 364 | 144 | 323 | 104 | 134 | 308 | 168 | 304 | 162 | 387 | 134 | 168 | 174 | 126 | 269 |
| YV150 1-2 | C9 | 186 | 285 | 137 | 161 | 191 | 364 | 144 | 323 | 104 | 134 | 308 | 168 | 304 | 162 | 387 | 134 | 168 | 174 | 126 | 269 |
| YV150 1-2 | C22 | 188 | 285 | 137 | 161 | 191 | 364 | 144 | 323 | 104 | 134 | 308 | 168 | 304 | 162 | 387 | 134 | 168 | 174 | 126 | 269 |
| YV150 1-2 | C2 | 186 | 285 | 137 | 161 | 191 | 364 | 144 | 323 | 104 | 134 | 308 | 168 | 304 | 162 | 387 | 134 | 168 | 174 | 126 | 269 |
| YV150 1-2 | C4 | 186 | 285 | 137 | 161 | 191 | 364 | 144 | 323 | 104 | 134 | 308 | 168 | 304 | 165 | 387 | 134 | 168 | 174 | 126 | 269 |
| YV150 1-2 | C5 | 186 | 285 | 137 | 161 | 191 | 364 | 144 | 323 | 104 | 134 | 308 | 168 | 304 | 162 | 387 | 134 | 168 | 174 | 126 | 269 |
| YV150 1-2 | C6 | 186 | 285 | 137 | 161 | 191 | 364 | 144 | 323 | 104 | 134 | 308 | 168 | 304 | 162 | 387 | 134 | 168 | 174 | 126 | 269 |
| YV150 1-2 | N1 | 188 | 285 | 137 | 161 | 191 | 364 | 144 | 323 | 104 | 134 | 308 | 168 | 304 | 162 | 387 | 132 | 168 | 174 | 126 | 269 |
| YV150 1-2 | N2 | 186 | 285 | 137 | 161 | 191 | 364 | 144 | 323 | 104 | 134 | 308 | 168 | 304 | 162 | 387 | 132 | 168 | 174 | 126 | 269 |
| YV150 1-2 | N3 | 186 | 285 | 137 | 161 | 191 | 364 | 144 | 323 | 104 | 134 | 308 | 168 | 304 | 162 | 387 | 132 | 168 | 174 | 126 | 269 |
| YV150 1-2 | N4 | 186 | 285 | 137 | 161 | 191 | 364 | 144 | 323 | 104 | 134 | 308 | 168 | 304 | 162 | 387 | 132 | 168 | 174 | 126 | 269 |
| YV150 1-2 | N5 | 186 | 288 | 137 | 164 | 191 | 364 | 144 | 323 | 104 | 134 | 308 | 168 | 304 | 162 | 387 | 132 | 168 | 174 | 126 | 269 |
| YV150 1-2 | N6 | 186 | 285 | 137 | 161 | 191 | 364 | 144 | 323 | 104 | 134 | 308 | 168 | 304 | 162 | 387 | 132 | 168 | 174 | 126 | 269 |
| YV150 1-2 | N7 | 186 | 285 | 137 | 161 | 191 | 364 | 144 | 330 | 104 | 134 | 308 | 168 | 304 | 162 | 387 | 132 | 168 | 178 | 126 | 269 |
| YV150 1-2 | N10 | 186 | 285 | 137 | 161 | 191 | 364 | 144 | 323 | 104 | 134 | 308 | 168 | 304 | 162 | 387 | 132 | 168 | 174 | 126 | 269 |
| YV150 1-2 | N11 | 186 | 288 | 137 | 161 | 191 | 364 | 144 | 326 | 104 | 134 | 308 | 168 | 304 | 162 | 390 | 132 | 168 | 174 | 126 | 269 |
